# Estimation of the proteomic cancer co-expression sub networks by using association estimators

**DOI:** 10.1371/journal.pone.0188016

**Published:** 2017-11-16

**Authors:** Cihat Erdoğan, Zeyneb Kurt, Banu Diri

**Affiliations:** 1 Department of Computer Engineering, Namik Kemal University, Tekirdag, Turkey; 2 Department of Integrative Biology and Physiology, University of California Los Angeles, Los Angeles, California, United States of America; 3 Department of Computer Engineering, Yildiz Technical University, Istanbul, Turkey; Kyushu University, JAPAN

## Abstract

In this study, the association estimators, which have significant influences on the gene network inference methods and used for determining the molecular interactions, were examined within the co-expression network inference concept. By using the proteomic data from five different cancer types, the hub genes/proteins within the disease-associated gene-gene/protein-protein interaction sub networks were identified. Proteomic data from various cancer types is collected from The Cancer Proteome Atlas (TCPA). Correlation and mutual information (MI) based nine association estimators that are commonly used in the literature, were compared in this study. As the gold standard to measure the association estimators’ performance, a multi-layer data integration platform on gene-disease associations (DisGeNET) and the Molecular Signatures Database (MSigDB) was used. Fisher's exact test was used to evaluate the performance of the association estimators by comparing the created co-expression networks with the disease-associated pathways. It was observed that the MI based estimators provided more successful results than the Pearson and Spearman correlation approaches, which are used in the estimation of biological networks in the weighted correlation network analysis (WGCNA) package. In correlation-based methods, the best average success rate for five cancer types was 60%, while in MI-based methods the average success ratio was 71% for James-Stein Shrinkage (Shrink) and 64% for Schurmann-Grassberger (SG) association estimator, respectively. Moreover, the hub genes and the inferred sub networks are presented for the consideration of researchers and experimentalists.

## Introduction

In bioinformatics, various approaches are leveraged to understand the molecular perturbations observed on cells or tissues caused by a disease, such as cancer, autism, diabetes, Alzheimer's. One of these approaches is inferring the gene networks, which can illustrate the gene-gene and protein-protein interactions from an expression dataset to understand the cell physiology and disease pathogenesis and to estimate the genome-wide working mechanism of proteins and genes [1,2]. In this study, effects of the association estimators on the network inference methods, which are frequently utilized in bioinformatics studies to detect molecular structures related to a given disease, were evaluated via the proteomic data of five different cancer types generated by TCPA [3]. The five cancer types used in this study are among the most common types according to the recently published results by the American Cancer Society [4].

In previous studies, both the synthetic and real gene expression data sets obtained from the microarray assays are mainly used to analyze the association estimators’ effect on the gene network inference techniques. Olsen et al. [5] evaluated the performance of Pearson, Spearman, Empirical, Miller-Madow (MM), and Shrink association estimators on three network inference algorithms (Algorithm for the Reconstruction of Accurate Cellular Networks–ARACNE [6], Context-Likelihood of Relatedness–CLR [7], Network inference with maximum relevance/minimum redundancy feature selection–MRNET [8]) by using synthetic and real microarray data sets. In addition, the Empirical, MM and Shrink estimators were used to examine the equal width (EW) and equal frequency (EF) discretization methods’ effects on the performance of the association estimators [5]. As a result, MRNET with the Spearman and CLR with the Pearson yielded more successful results on the synthetic dataset, and also significant results were obtained on the real dataset, as well [5]. Simoes and Streib [9] used the MM, ML, Shrink and SG association estimators with three different discretization methods (EW, EF, global equal width—GEW) together with The Conservative Causal Core NETwork (C3NET) [10] network inference algorithm by using the synthetic gene microarray data sets to examine the effects of both the estimators and the discretization methods on the network inference algorithms. They found that the MM estimator outperformed the other estimators when used with the EW discretization method. Daub et al. [11], investigated the performances of the B-spline (BS) and Kernel Density Estimator (KDE) methods on large-scale gene expression data sets. As a result, they found that the performance of the BS estimator varied for different spline values. The order of success is as follows BS (spline order = 3) > KDE > BS (spline order = 1). Kurt et al. [12], used two synthetic and two biological data sets to evaluate 14 association estimators with 3 different network inference algorithms. Also they observed that the influences of the Copula Transform (CT) pre-processing operation on the performance of the association estimators. B-spline, Pearson-based Gaussian and Spearman-based Gaussian estimators are observed as the best performing ones among all. Also CT operation increased inference performances of the estimators for synthetic datasets.

Network inference methods have been used in multiple studies on different cancer types. Şenbabaoğlu et al. [13] analyzed the protein expression data, which was obtained from TCPA, from 3467 patients with 11 different types of cancer by using 13 different network inference methods. The most successful network inference method was varying based on the cancer type. Madhamshettiwar et al. [14] searched the biological mechanisms related to the ovarian cancer using 7 different network inference methods.

WGCNA [15] is one of the most commonly used gene network inference and clustering approaches and is used to find highly correlated gene/protein modules (cluster, sub-network) related to a given disease and hub genes/proteins with high connectivity in these modules. WGCNA has been used to analyze gene/protein expression data from brain cancer [16], yeast cell cycle [17], mouse genetics [18,19], diabetes [20] and many other diseases and complex traits.

It has been reported that the correlation-based methods are not sufficient to estimate the non-linear relationships between the cell molecules such as the complex molecular interactions related to a cancer type [21]. Many algorithms have been developed to solve this problem by using mutual information based methods [6–8,10].

We proposed a system integrating the MI-based association estimators with the co-expression network clustering technique to identify the sub networks associated with the diseases. Leveraging the MI-based estimators in WGCNA instead of the correlation-based ones, which are the default and only options presented in the WGCNA, can capture both linear and non-linear interactions between the protein-protein pairs and improves the estimation of disease-associated molecular mechanisms and interactions, while the correlation-based ones can only capture the disease mechanisms based on the linear interactions. To estimate the disease associated sub networks and mechanisms, we leveraged the MI-based estimators within the *co-expression network methodology* different than the previous studies [13,14] that were interested in global networks and not focusing on the individual sub networks or co-expression modules. Also different discretization methods were used to estimate the association estimators that are widely used in gene network inference, and an evaluation framework was developed to analyze the as-obtained results unlike [13,14]. Besides, we identified the hub genes of the sub networks to reveal the potential key regulators of these sub networks, which were not searched in [6–8]. We observed that according to whole cancer datasets used in our study Shrink, SG, BS, MM and Shrink methods identified the most disease-relevant sub networks and genes with average precision scores of 0.85, 0.84, 0.59, 0.76 and 0.76 (for BRCA, GBM, LUSC, KIRC, and SKCM datasets), respectively. Moreover, unlike previous studies [5], [9], [11,12], in our study, five real cancer proteomic data sets that are obtained from TCPA were examined by biological network inference method along with nine different association estimators to compare the association estimators’ performance.

## Materials and methods

### Materials

Studies in bioinformatics field have gained a great momentum along with the developments in high-throughput techniques. One of these techniques is the reverse phase protein array (RPPA) [22] technology, which is a high-throughput antibody-based technique and it supplies protein expression data for proteomics research. Sheehan et al. [23] performed an analysis on ovarian cancer data obtained by RPPA. TCPA has obtained protein expression data from numerous cell line and tumor samples using RPPA technique. The detailed information about the process of preparing the data set before the analysis is given in [3].

The RPPA data used in this study was downloaded from **Download** section (level 4) in TCPA’s web site [24]. It is comprised of proteomic expression data of 2230 cancer patients in 5 cancer types. There are more than 218 antibodies for each patient. To obtain the individual gene-protein matching (S1 Table) of the data, a process was performed after taking the relevant information from **My Protein** section of TCPA’s [24] web site. The file containing the expression data of the selected antibodies (proteins) for each cancer type is given (S2–S6 Tables). The type of the cancers, their sample sizes, number of proteins (antibodies), number of selected antibodies and their abbreviations are listed in Table 1.

**Table 1:** The type of cancers and their sample sizes (Level 4).

	The cancer tumor type	Cancer abbreviation	Number of samples	Number of proteins	Number of selected proteins
1	Breast invasive carcinoma	BRCA	901	224	173
2	Glioblastoma multiforme	GBM	205	223	173
3	Lung squamous cell carcinoma	LUSC	325	237	170
4	Clear cell kidney carcinoma	KIRC	445	233	169
5	Skin Cutaneous Melanoma	SKCM	354	223	173

Furthermore, to evaluate the performances of the association estimators, DisGeNET and MSigDB were used as gold standards. DisGeNET curates the gene-disease and genetic variant-disease associations that were reported in previous studies and stored in multiple publically available data sources such as UNIPROT database, the Comparative Toxicogenomics Database, GWAS catalog, and also it contains 429,036 associations between 17,381 genes and 15,093 diseases [25,26]. In MSigDB, there are 17,779 gene sets in total, from 8 different collections, namely hallmark gene sets, positional gene sets, curated gene sets, motif gene sets, computational gene sets, Gene Ontology (GO) gene sets, oncogenic gene sets, and immunologic gene sets [27].

Based on each cancer type, the search terms used to obtain the data, the Unified Medical Language System—Concept Unique Identifiers (UMLS-CUIs), number of the genes obtained from DisGeNET with a number of supporting publications (PMIDs) ≥ 2 and the overlapped number of the genes between the selected cancer dataset and DisGeNET are given in Table 2. The gene level analysis was performed by using the relevant cancer-related DisGeNET data.

**Table 2:** Information of data from DisGeNET.

	Cancer Type	Search terms on DisGeNET	UMLS-CUIs	DisGeNET gene number (PMIDs> = 2)	Overlapped Gene number between TCPA Dataset and DisGeNET
1	BRCA	Breast Cancer	C0346153	52	15
2	GBM	Glioblastoma Multiforme	C1621958	147	28
3	LUSC	Squamous cell carcinoma of lung	C0149782	41	7
4	KIRC	Kidney Diseases	C0022658	264	14
5	SKCM	Cutaneous Melanoma	C0151779	104	25

UMLS-CUIs is abbreviation of Unified Medical Language System—Concept Unique Identifiers and PMIDs is the number of supporting publications.

By using the selected disease genes from DisGeNET for each cancer type, the top associated 100 biological pathways were identified from the BioCarta, KEGG, and Reactome databases with a false discovery rate (FDR)<0.05 by using MSigDB online tool. FDR score is the adjusted version of the raw p-value for the multiple hypothesis testing. Here, Bonferroni correction was used to correct the raw p-values [28], in which the p-values are multiplied by the number of hypotheses that were tested. The pathway level analysis was performed by using the top relevant biological pathways of the disease genes obtained from the DisGeNET.

### Methods

In this study, the association scores between the proteins were calculated by using Spearman [5], Pearson [5], Kendall Tau (Kendall) [29] correlation methods. BS [11], Empirical [30], KDE [31], MM [32], Shrink [33], and SG [34] MI-based association estimators were also used in our analyses. Moreover, the influence of the discretization methods (EW, EF, GEW) on the Empirical, MM, Shrink, and SG estimators’ inference performances was also examined. Details of the association estimator and the discretization methods used in this study can be found in [5,12,33,35]. All these methods were used as an alternative to the correlation-based *adjacency* function of WGCNA package that is used to calculate the Pearson and Spearman correlation scores between the protein pairs. We compared the MI-based and correlation-based methods within the co-expression network concept and evaluated their disease relevance in both pathway and gene-level.

The WGCNA steps were performed as follows;

- Firstly, the association matrix (*adjacency*) of the proteins in the data set is calculated using the protein expression data.
- A network-related similarity matrix is obtained by using a topological overlap measure (TOM) [36], which has been successfully applied in different studies [37] in the literature, to identify the heavily interrelated protein clusters (*TOMsimilarity* measure).
- The obtained similarity matrix is subtracted from one (1-TOM) to identify a dissimilarity measure. From the dissimilarity matrix, a clustering tree is formed to identify the cluster of related proteins via hierarchical clustering (*hclust*).
- To find highly correlated gene/protein co-expression modules in the generated clustering tree of the gene networks, we used the dynamic branch cut methods [38] (*cutreeDynamic*) with different parameters (see S7 Table). Finally, gene/protein with the highest connectivity in each module (hub gene) is determined. (*chooseOneHubInEachModule*).

In this study, we used one-tailed version of the Fisher's exact test (FET) [39], which is identical to the hypergeometric test to calculate the P-values representing the association of the co-expression modules with the given cancer type. A hypergeometric test is adopted from [40], whose distribution (used to calculate P-value) is given in (1) where n and k as integer, ${(}_{k}^{n})$ is the binomial coefficient, I is the number of inferred genes/proteins of network inference method, V is the number of genes/proteins in the DisGeNET database to use for verification, O is the number of overlap between I and V. AGP (all genes/proteins) is the number of all known genes/proteins for human genome.

$$P(O)=\frac{\left(\frac{I}{O})(\frac{AGP-I}{V-O}\right)}{\left(\frac{AGP}{V}\right)}=\frac{I!\;(AGP-I)!V!\;(AGP-V)!}{O!\;(I-O)!\;(V-O)!\;(AGP-I-V+O)!AGP!}$$ (1)

**Fig 1:**
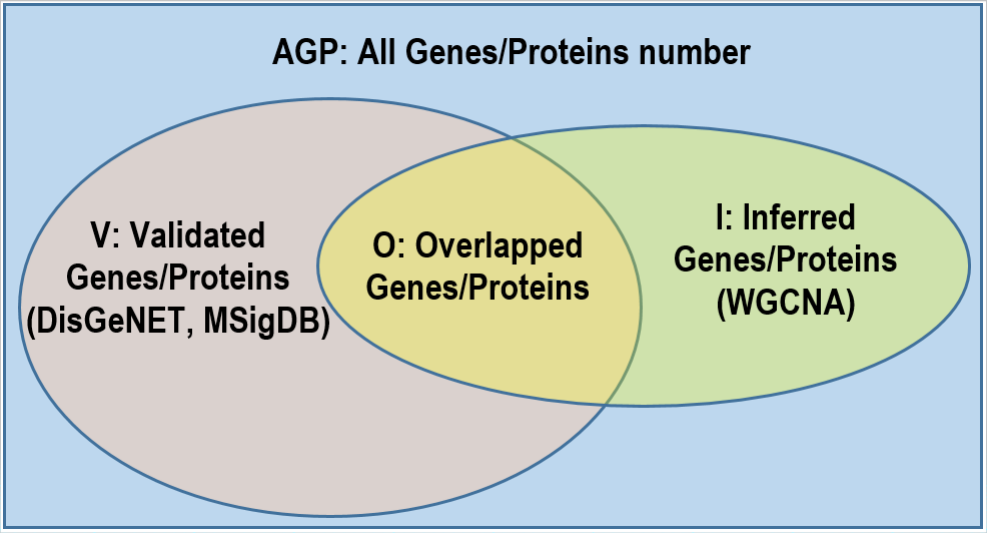
Overlap analysis with FET. Here, *I* is the count of inferred genes/proteins of network inference method (e.g., WGCNA). *V* is the count of genes/proteins in the literature database (e.g., DisGeNET, MSigDB) to search for verification. O is the number of overlap between *I* and *V*. *AGP* is the count of all genes/proteins in the literature.

If the calculated P-value is less than 0.05, the overlapped proteins between the inferred modules and disease-related DisGeNET genes and their relevant pathways, are considered less likely to be random and these modules are more likely to be disease-associated and biologically interesting. Fig 1 and Table 3 are illustrated to provide a better understanding for the use of FET for the overall assessment of the association estimators’ performances. As given in Fig 1, based on the given two clusters (e.g., inferred and validated genes/proteins), it was determined whether the overlap is statistically significant according to all genes/proteins (AGP) in the literature. Recent studies have revealed that the number of genes in the human genome is around 19,000 [41] and we used this value as AGP in our study. Table 3 summarizes the regions shown in Fig 1 and is used as an input in the FET.

**Table 3:** FET parameters in gene level.

	Inferred Genes/Proteins (in the literature)	Inferred Genes/Proteins (not in the literature)
**Genes/Proteins (Inferred)**	*O*	*I–O*
**Genes/Proteins (Not inferred)**	*V—O*	*AGP–I–V + O*

*I* is the count of inferred genes/proteins of network inference method. *V* is the count of genes/proteins in the literature database to search for verification. *O* is the number of overlap between *I* and *V*. AGP is the count of all genes/proteins in the literature.

Ultimate assessment metric used in the evaluation process is the *precision* score:

$$p=\frac{TP}{TP+FP}$$ (2)

where *p* is precision, *TP* is number of the true positives, *FP* is number of the false positives. TPs denote the modules which satisfying the required conditions in the pathway-level or gene-level analysis (P-value<0.05). FPs denote the number of modules which are not satisfying the required conditions in the pathway-level or gene-level analyses. The required conditions are described in Proposed Framework section.

#### Association estimators used in the reconstruction process of the co-expression networks

For the analyses, the *build*.*mim* function from the **minet** [35], the *obtain*.*mim* function from the **DepEst** [42] and the *chooseOneHubInEachModule*, *adjacency*, *TOMsimilarity*, *cutreeDynamic* and *hclust* functions from the **WGCNA** packages were used. The selected association estimators are indicated by the specific parameter values provided to the *build*.*mim* and *obtain*.*mim* functions.

#### Proposed framework

**Fig 2:**
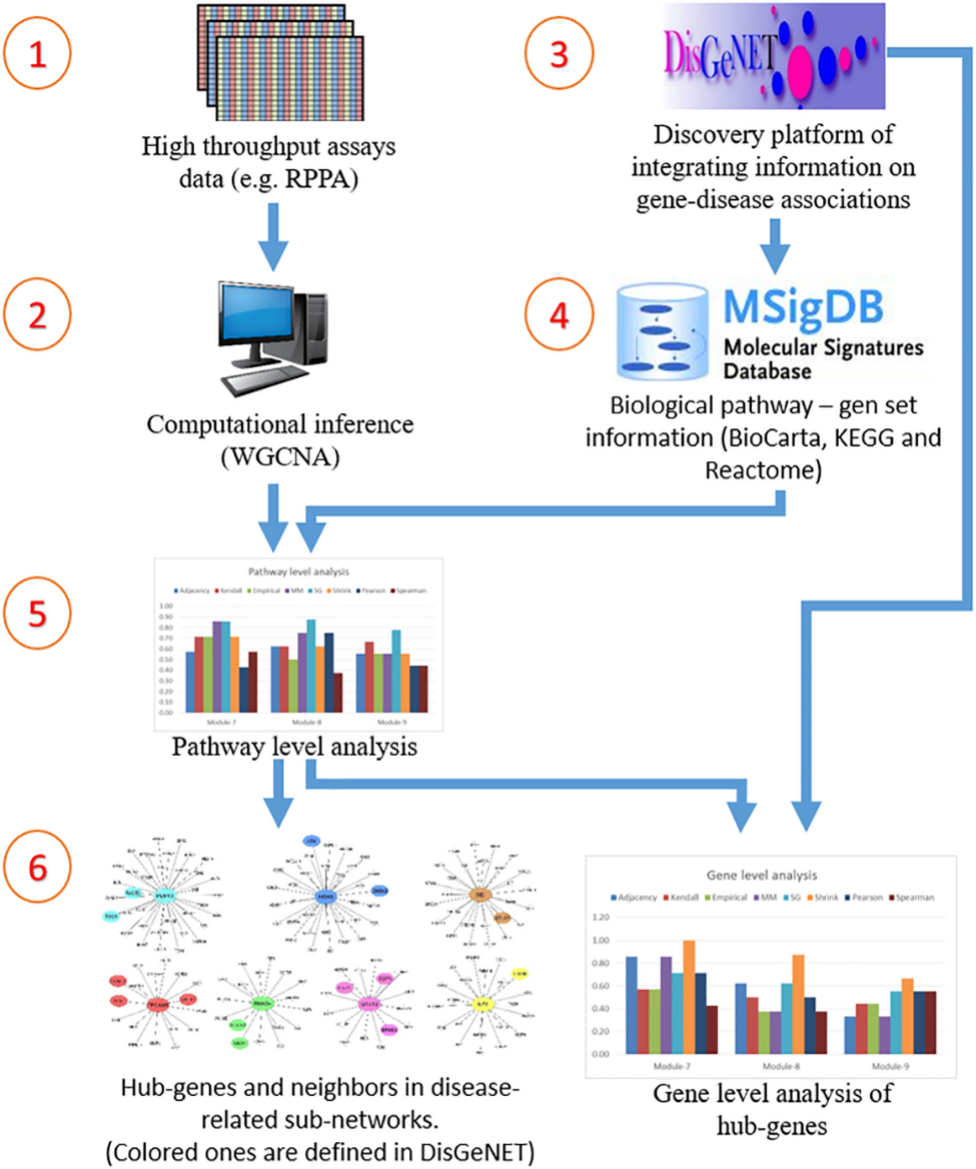
Proposed framework.

In this section, the proposed framework to analyze the performances of the association estimators is illustrated in Fig 2.

**Fig 3:**
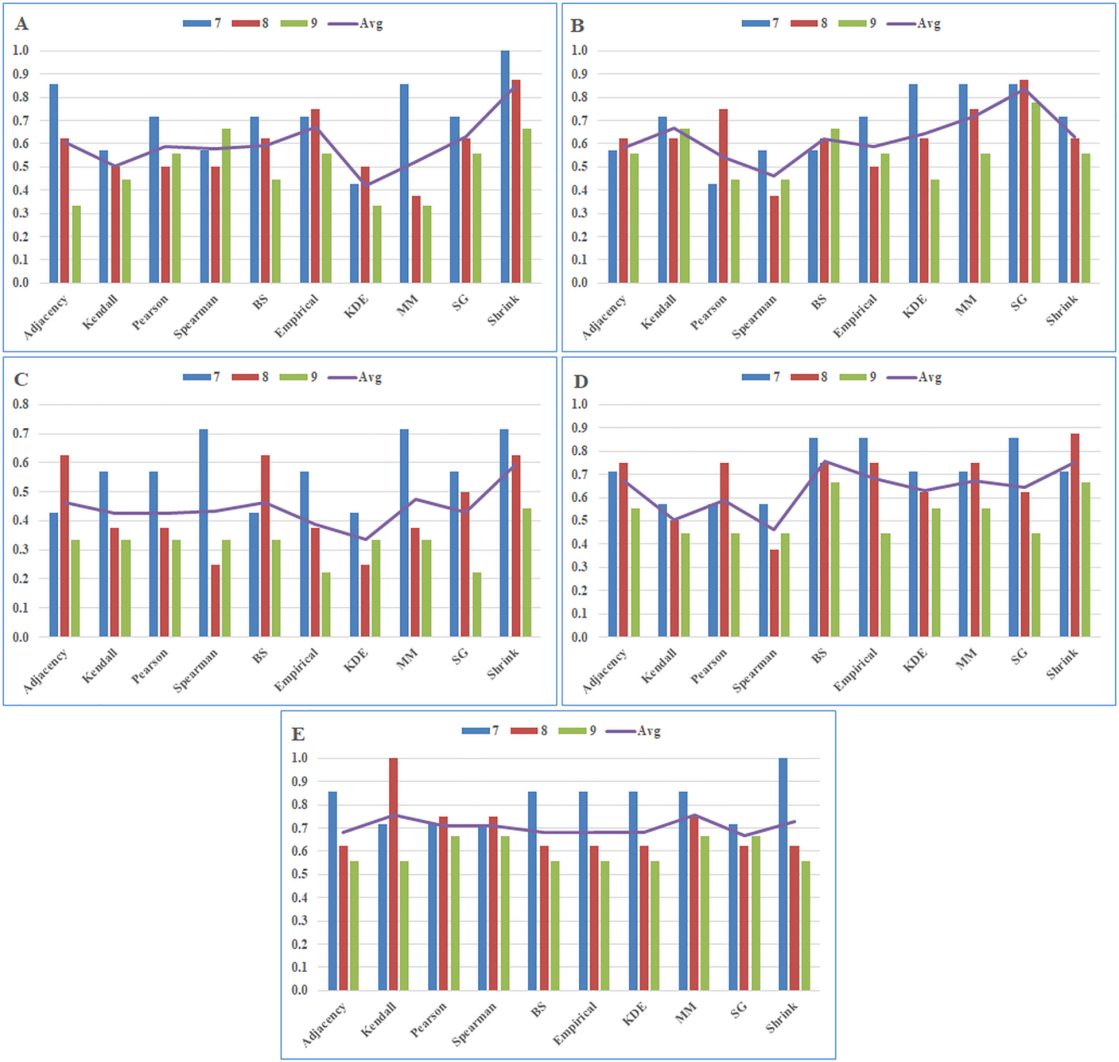
Pathway level analysis results by module number for each cancer type. Precision scores of A) BRCA, B) GBM, C) KIRC, D) LUSC, and E) SKCM datasets. The abbreviations: BS, B-spline; KDE, Kernel Density Estimator; MM, Miller-Madow; SG, Schurmann-Grassberger; Shrink, James-Stein Shrinkage. Avg is the average performance score of the association estimators according to the constructed module sizes.

**Fig 4:**
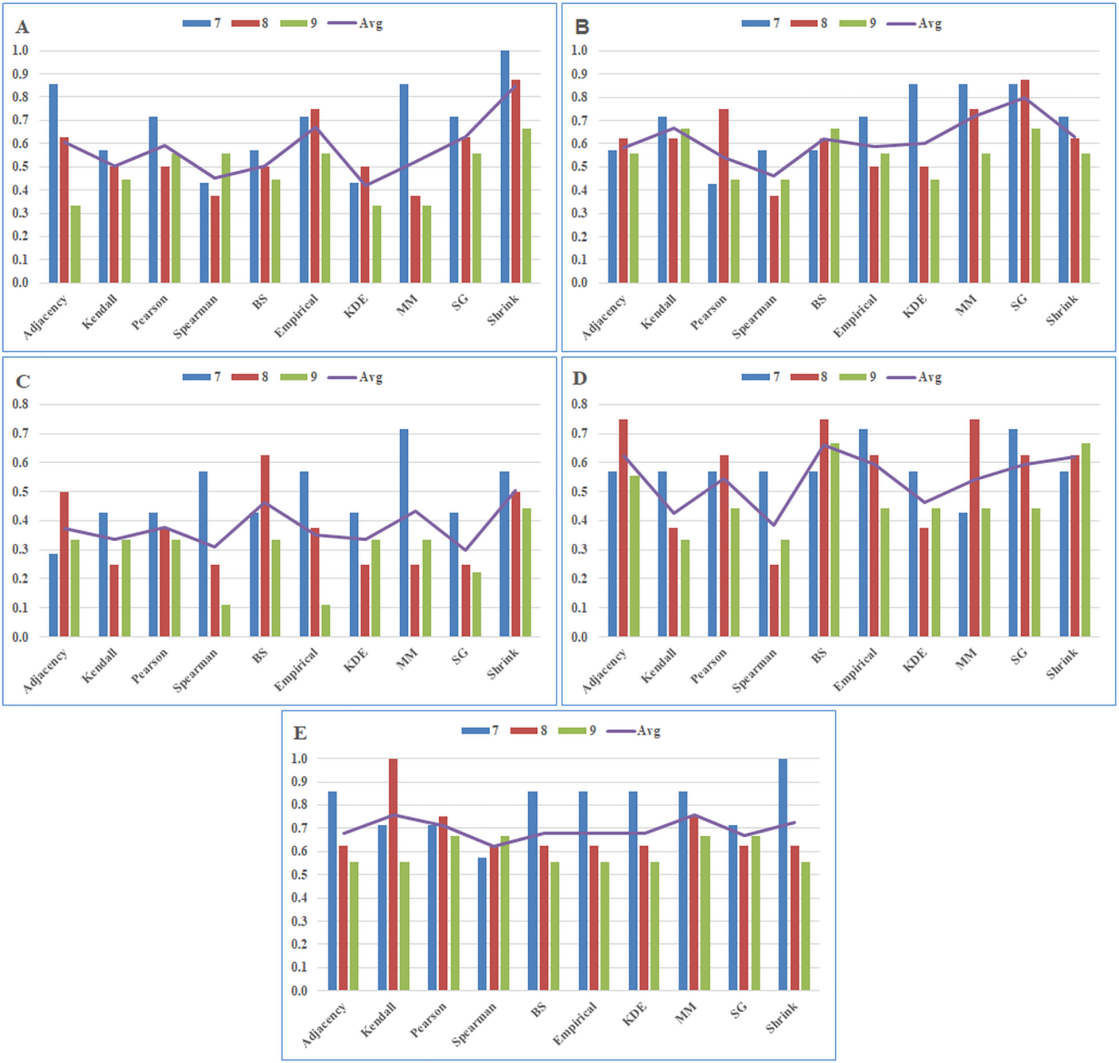
Gene level analysis results by module number for each cancer type (P-value ≤ 0.05). Precision scores of A) BRCA, B) GBM, C) KIRC, D) LUSC, and E) SKCM datasets. The abbreviations: BS, B-spline; KDE, Kernel Density Estimator; MM, Miller-Madow; SG, Schurmann-Grassberger; Shrink, James-Stein Shrinkage. Avg is the average performance score of the association estimators according to the constructed module sizes.

**Fig 5:**
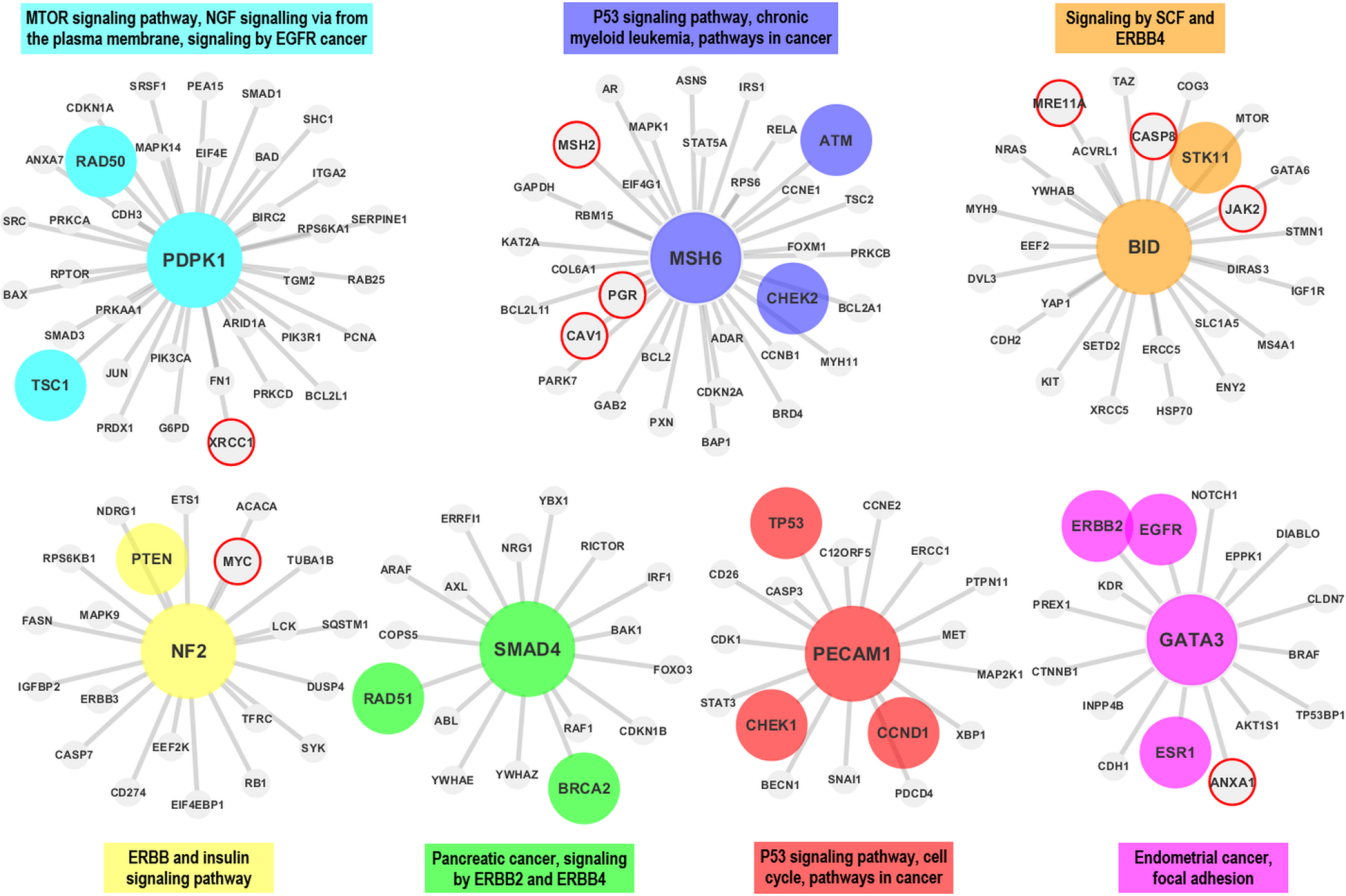
The hub genes and neighbors in the disease-related sub-networks obtained by the most successful Shrink method (in terms of precision score) on BRCA dataset. *MSH6* and *GATA3* are validated in one study according to DisGeNET. *PDK1* and *SMAD4* genes (proteins) were also shown to be associated with the BRCA in multiple studies though they were not reported in the DisGeNET. The genes registered in DisGeNET and experimentally confirmed for the diseases, are shown with colored and larger nodes. Among those, genes that are not colored but have a red frame have a PMID value of one. There is no entry in DisGeNET for the grey colored nodes.

**Fig 6:**
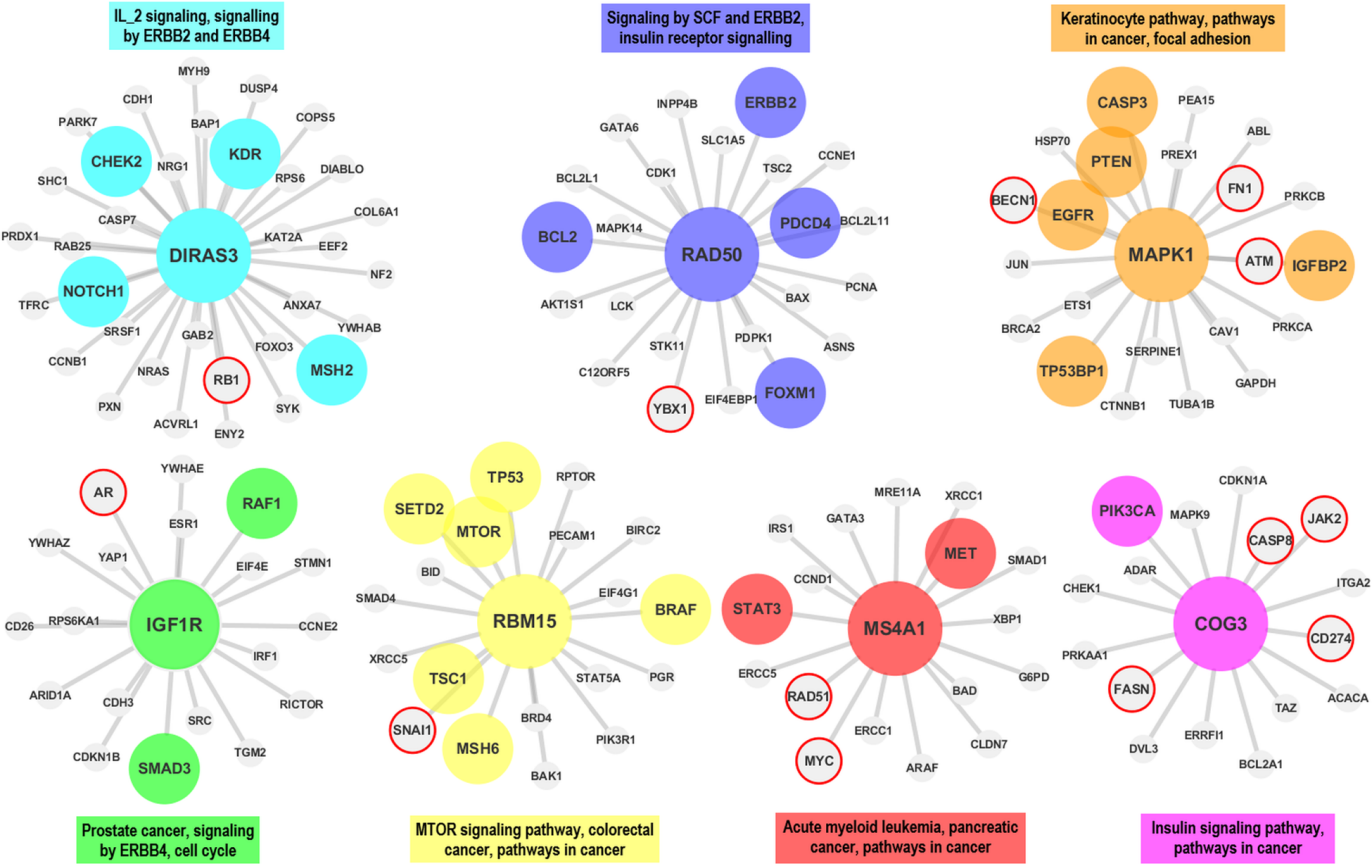
The hub genes and neighbors in the disease-related sub-networks obtained by the most successful SG method (in terms of precision score) on GBM dataset. *IGF1R* is validated in one study according to DisGeNET. In recent study, *RAD50* gene (protein) was also shown to be associated with the GBM, though it was not reported in the DisGeNET. The genes registered in DisGeNET and experimentally confirmed for the diseases, are shown with colored and larger nodes. Among those, genes that are not colored but have a red frame have a PMID value of one. There is no entry in DisGeNET for the grey colored nodes.

**Fig 7:**
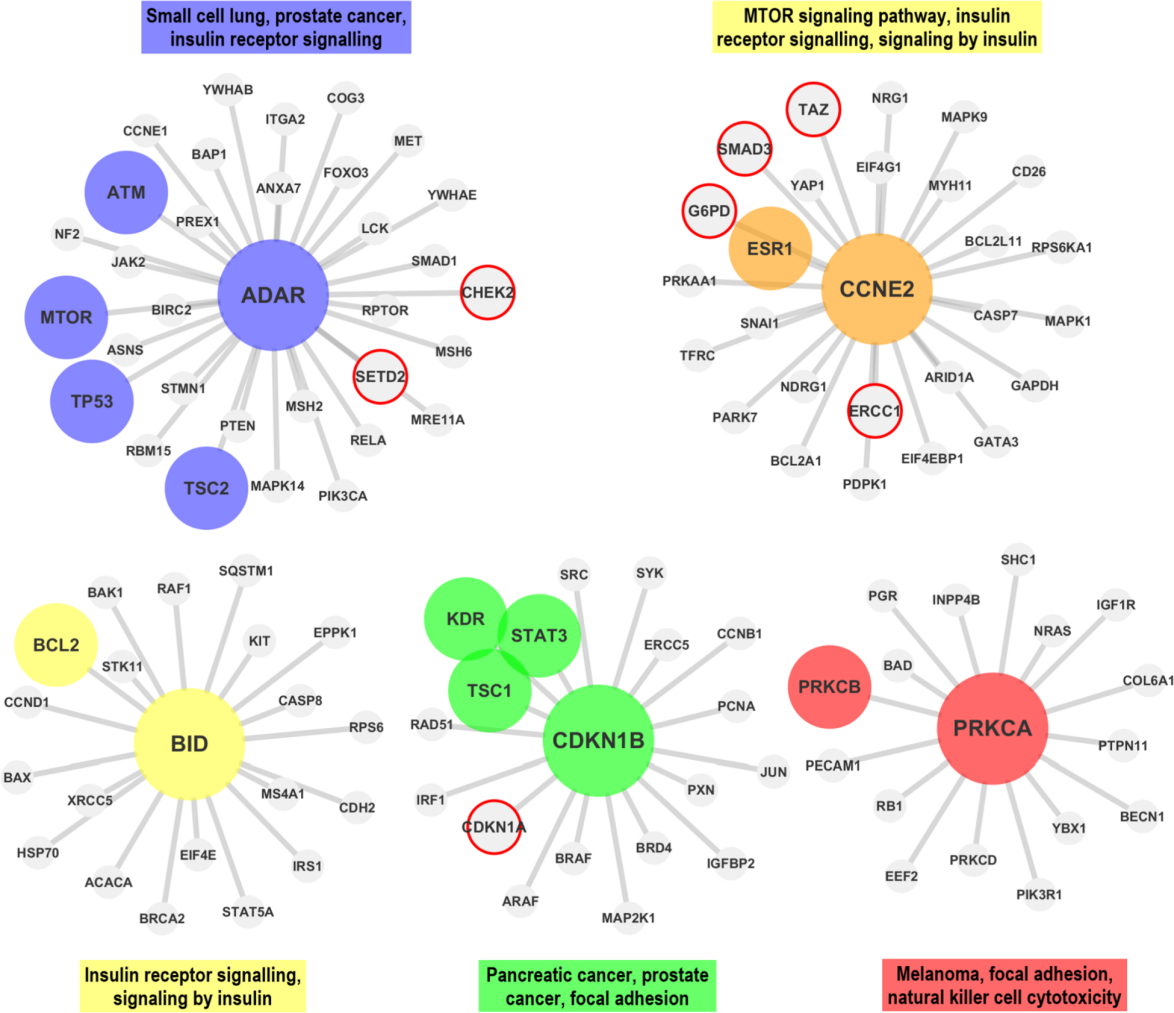
The hub genes and neighbors in the disease-related sub-networks obtained by the most successful Shrink method (in terms of precision score) on KIRC dataset. The genes registered in DisGeNET and experimentally confirmed for the diseases, are shown with colored and larger nodes. Among those, genes that are not colored but have a red frame have a PMID value of one. There is no entry in DisGeNET for the grey colored nodes.

**Fig 8:**
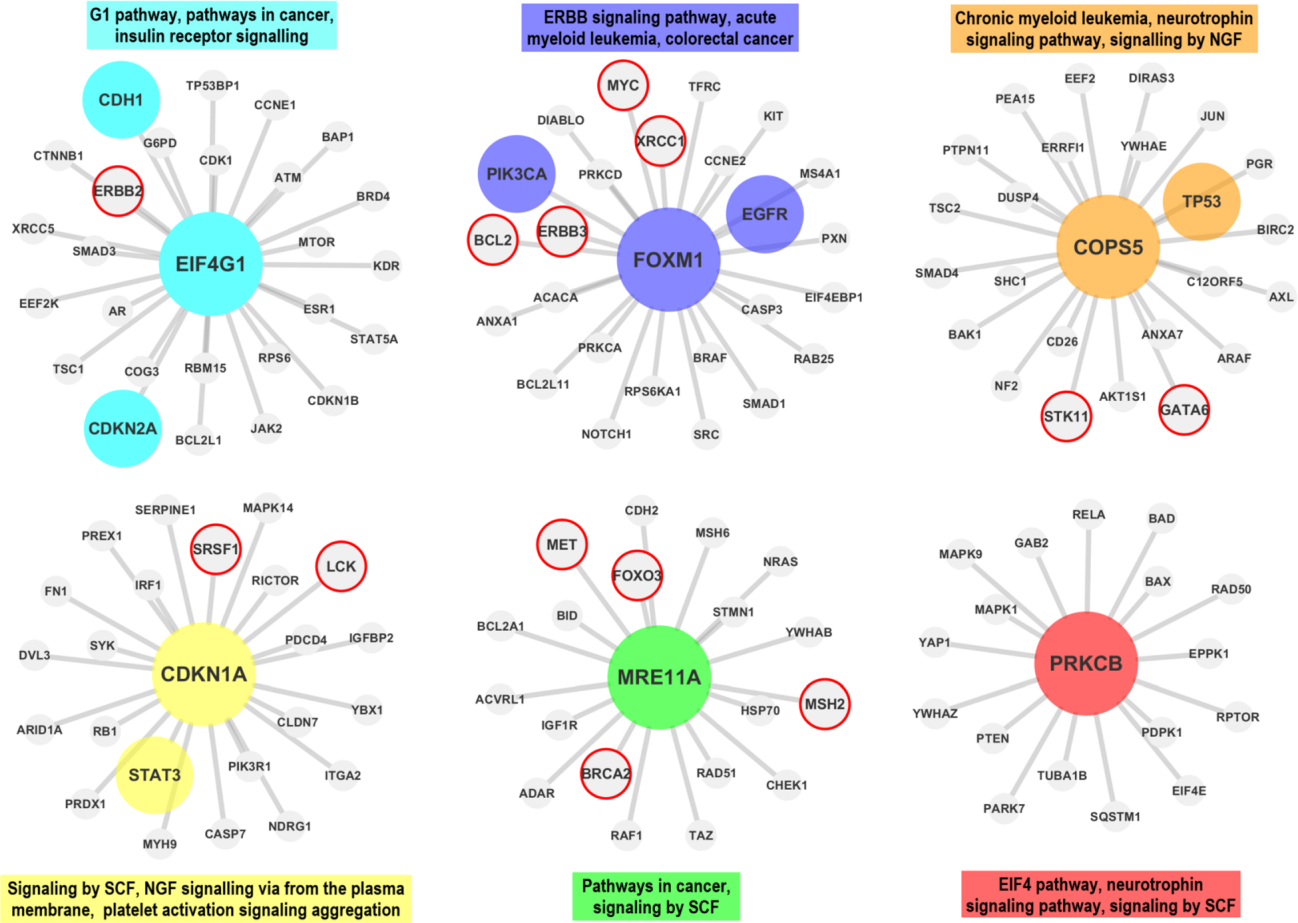
The hub genes and neighbors in the disease-related sub-networks obtained by the most successful BS method (in terms of precision score) on LUSC dataset. *EIF4G1* is validated in multiple studies according to DisGeNET. In recent studies, *FOXM1* and *CDKN1A (P21)* genes (proteins) were also shown to be associated with the LUSC, though they were not reported in the DisGeNET. The genes registered in DisGeNET and experimentally confirmed for the diseases, are shown with colored and larger nodes. Among those, genes that are not colored but have a red frame have a PMID value of one. There is no entry in DisGeNET for the grey colored nodes.

**Fig 9:**
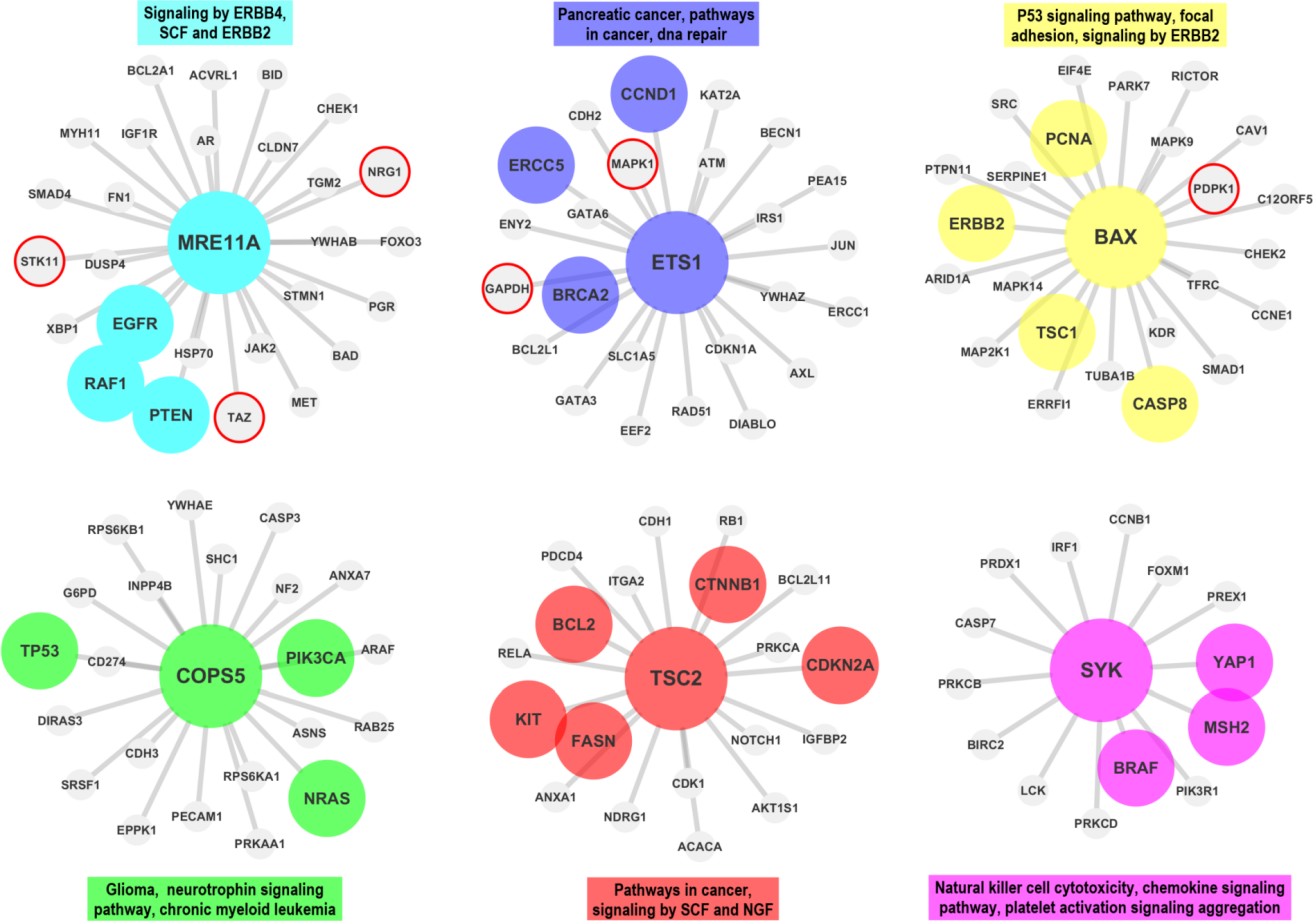
The hub genes and neighbors in the disease-related sub-networks obtained by the most successful MM method (in terms of precision score) on SKCM dataset. In recent studies, *ETS1* gene (protein) was also shown to be associated with the SKCM, though it was not reported in the DisGeNET. The genes registered in DisGeNET and experimentally confirmed for the diseases, are shown with colored and larger nodes. Among those, genes that are not colored but have a red frame have a PMID value of one. There is no entry in DisGeNET for the grey colored nodes.

1. In the first step the proteomic data is downloaded from TCPA and prepared for the analysis (see *Materials* section).
2. In the second step, co-expression networks and modules were created using WGCNA as described in *Methods* section. The interaction score matrix to be used in the subsequent steps was calculated by using the adjacency function and other association estimators provided by DepEst [42] and minet [35] packages. In addition, parameters that generate at least 7 modules (sub-networks) for each association estimator were found by using certain parameters (see S7 Table). 7, 8, and 9 modules are generated from the co-expression networks for each association estimator from each dataset. The reason why the module numbers are selected as 7-8-9 is shown in (S1 and S2 Figs) and details are explained in S1 Text. The modules created with the parameters listed in S8 Table were used in the comparison of the association estimators.
3. In the 3rd step, the genes that were confirmed to be related with the given cancer type in at least two different studies (PMIDs ≥ 2) were obtained for each cancer type by using DisGeNET web page [43].
4. In this step, by using the MSigDB web page [44], we identified the top 100 biological pathways from the BioCarta, KEGG and Reactome databases, that are significantly associated with the disease-related gene list with a FDR q-value <0.05.
5. Pathway-level analysis by FET: In this step, overlapping ratios and corresponding *P*-values of the association between the pathways obtained at the 4th step and the modules identified in step 2 were found by using FET. To correct the raw P-values for the multiple hypothesis testing, the FDR q-value was calculated by multiplying the P-value by 100 since we selected the top 100 pathways associated with the DisGeNET genes in the previous step. To evaluate our findings in the pathway level, WGCNA modules containing at least 5 shared genes with at least two of the disease-associated pathways found in Step 4 with an FDR q-value <0.05 were considered as a successful hit (*TP*) in terms of disease relevance. The reason why at least 2 pathways and 5 genes are selected as cut-off is shown in S3 Fig and details are explained in S2 Text. Number of the modules satisfying these conditions are divided by the total number of the modules *(TP+FP)* to obtain the *pathway level* performance scores (*precision*) of the association estimators. The precision ratios at the pathway level are given in Table 4 and Fig 3 by module numbers for each association estimator.
6. Gene-level analysis by FET: To evaluate our findings in gene level, the overlapping ratios between the genes in modules passing the overlap analysis test at the pathway level overlap analysis and the genes obtained from DisGeNET were calculated via FET, and the resulting modules which have p-values lower than 0.05 were considered as significantly associated (*TP*) with the corresponding cancer type. By dividing the number of modules that satisfy these conditions by the total number of modules (*TP+FP*), we obtained the *gene level* performance scores (*precision*) of the association estimators estimator (see S9 Table for details of the gene-level analysis scores by FET). The precision scores at the gene level are given in Table 5 by module numbers for each association estimator, and the graphical representation of the hub genes of the significantly disease-relevant modules is given in Fig 4. In this step, the hub genes within the disease-associated sub-networks, which are identified by the most successful estimator of step 5, and the neighboring genes that are located at the center of these hub genes are illustrated in Figs 5–9 for BRCA, GBM, KIRC, LUSC, SKCM cancer type, respectively. The hub genes and the genes that have been experimentally confirmed to be associated with the disease and reported in DisGeNET within the hub gene neighborhood are illustrated with colored and larger nodes in Figs 5–9. The genes that are not colored but have a red frame in Figs 5–9 have a PMID value of one. There is no entry in DisGeNET for the grey colored nodes. Also, the top three pathways, to which each module is related, are given above or below the relevant module to annotate each module.

**Table 4:** Pathway level precision ratios by module numbers for each cancer type.

	BRCA				GBM				KIRC				LUSC				SKCM			
	7	8	9	Avg	7	8	9	Avg	7	8	9	Avg	7	8	9	Avg	7	8	9	Avg
Adjacency	0.86	0.63	0.33	0.61	0.57	0.63	0.56	0.58	0.43	0.63	0.33	0.46	0.71	0.75	0.56	0.67	0.86	0.63	0.56	0.68
Kendall	0.57	0.50	0.44	0.51	0.71	0.63	0.67	0.67	0.57	0.38	0.33	0.43	0.57	0.50	0.44	0.51	0.71	1.00	0.56	**0.757**
Pearson	0.71	0.50	0.56	0.59	0.43	0.75	0.44	0.54	0.57	0.38	0.33	0.43	0.57	0.75	0.44	0.59	0.71	0.75	0.67	0.71
Spearman	0.57	0.50	0.67	0.58	0.57	0.38	0.44	0.46	0.71	0.25	0.33	0.43	0.57	0.38	0.44	0.46	0.71	0.75	0.67	0.71
BS	0.71	0.63	0.44	0.59	0.57	0.63	0.67	0.62	0.43	0.63	0.33	0.46	0.86	0.75	0.67	**0.76**	0.86	0.63	0.56	0.68
Empirical	0.71	0.75	0.56	0.67	0.71	0.50	0.56	0.59	0.57	0.38	0.22	0.39	0.86	0.75	0.44	0.68	0.86	0.63	0.56	0.68
KDE	0.43	0.50	0.33	0.42	0.86	0.63	0.44	0.64	0.43	0.25	0.33	0.34	0.71	0.63	0.56	0.63	0.86	0.63	0.56	0.68
MM	0.86	0.38	0.33	0.52	0.86	0.75	0.56	0.72	0.71	0.38	0.33	0.47	0.71	0.75	0.56	0.67	0.86	0.75	0.67	**0.758**
SG	0.71	0.63	0.56	0.63	0.86	0.88	0.78	**0.84**	0.57	0.50	0.22	0.43	0.86	0.63	0.44	0.64	0.71	0.63	0.67	0.67
Shrink	1.00	0.88	0.67	**0.85**	0.71	0.63	0.56	0.63	0.71	0.63	0.44	**0.59**	0.71	0.88	0.67	**0.75**	1.00	0.63	0.56	0.73

**Table 5:** Gene level precision ratios by module numbers for each cancer type (P-value <0.05).

	BRCA				GBM				KIRC				LUSC				SKCM			
	7	8	9	Avg	7	8	9	Avg	7	8	9	Avg	7	8	9	Avg	7	8	9	Avg
Adjacency	0.86	0.63	0.33	0.61	0.57	0.63	0.56	0.58	0.29	0.50	0.33	0.37	0.57	0.75	0.56	0.63	0.86	0.63	0.56	0.68
Kendall	0.57	0.50	0.44	0.51	0.71	0.63	0.67	0.67	0.43	0.25	0.33	0.34	0.57	0.38	0.33	0.43	0.71	1.00	0.56	**0.757**
Pearson	0.71	0.50	0.56	0.59	0.43	0.75	0.44	0.54	0.43	0.38	0.33	0.38	0.57	0.63	0.44	0.55	0.71	0.75	0.67	0.71
Spearman	0.43	0.38	0.56	0.45	0.57	0.38	0.44	0.46	0.57	0.25	0.11	0.31	0.57	0.25	0.33	0.38	0.57	0.63	0.67	0.62
BS	0.57	0.50	0.44	0.51	0.57	0.63	0.67	0.62	0.43	0.63	0.33	0.46	0.57	0.75	0.67	**0.66**	0.86	0.63	0.56	0.68
Empirical	0.71	0.75	0.56	0.67	0.71	0.50	0.56	0.59	0.57	0.38	0.11	0.35	0.71	0.63	0.44	0.59	0.86	0.63	0.56	0.68
KDE	0.43	0.50	0.33	0.42	0.86	0.50	0.44	0.60	0.43	0.25	0.33	0.34	0.57	0.38	0.44	0.46	0.86	0.63	0.56	0.68
MM	0.86	0.38	0.33	0.52	0.86	0.75	0.56	0.72	0.71	0.25	0.33	0.43	0.43	0.75	0.44	0.54	0.86	0.75	0.67	**0.758**
SG	0.71	0.63	0.56	0.63	0.86	0.88	0.67	**0.80**	0.43	0.25	0.22	0.30	0.71	0.63	0.44	0.59	0.71	0.63	0.67	0.67
Shrink	1.00	0.88	0.67	**0.85**	0.71	0.63	0.56	0.63	0.57	0.50	0.44	**0.51**	0.57	0.63	0.67	0.62	1.00	0.63	0.56	0.73

## Results

As shown in Table 4 and Fig 3, the rankings of the association estimators identified by the average performance score (Avg) are varying according to the constructed module sizes and the cancer type. In the pathway-level analysis, best performing methods based on the cancer type are as follows: Shrink for BRCA, SG for GBM, Shrink for KIRC, BS for LUSC, and MM for SKCM. In addition, although the most successful predictor varies for each data set, the MI based predictors outperformed other methods in BRCA, GBM, KIRC, LUSC datasets, however only in SKCM data set, MM estimator and Kendall correlation method were found to be successful with very close average precision scores according to the pathway level analysis.

According to the average precision scores for BRCA dataset that are given in Table 4, the ranking of the estimators is as follows: Shrink, Empirical, SG, Adjacency, BS, Pearson, Spearman, MM, Kendall and KDE. Here, Shrink method is the most accurate method in terms of identifying the disease-associated sub networks with a precision score of 0.85. Empirical, SG, Adjacency, BS, Pearson, Spearman methods follow this one with lower scores of 0.67, 0.63, 0.61, 0.59, 0.59, 0.58, respectively. Additionally, MM and Kendall methods gave comparatively lower scores of 0.52, 0.51, while KDE method gets the lowest score of 0.42.

Based on the average precision scores for GBM dataset (Table 4), the ranking of the success rate of the estimators is as follows: SG, MM, Kendall, KDE, Shrink, BS, Empirical, Adjacency, Pearson and Spearman. Here, SG method is the most accurate one in terms of identifying disease-relevant sub networks with a precision score of 0.84. MM, Kendall, KDE, Shrink, BS methods follow this one with lower scores of 0.72, 0.67, 0.64, 0.63, 0.62, respectively. Additionally, Empirical, Adjacency and Pearson methods concluded with comparatively lower scores of 0.59, 0.58, 0.54, while Spearman method gets the lowest score of 0.46.

According to the average precision scores for KIRC dataset that are given in Table 4, the ranking of the estimators is as follows: Shrink, MM, Adjacency, BS, Spearman, SG, Kendall, Pearson, Empirical and KDE. Here, Shrink method is identified as the most accurate one in terms of identifying disease associated sub networks with a precision score of 0.59. MM, Adjacency, BS, Spearman, SG, Kendall, Pearson methods follow this one with lower scores of 0.47, 0.46, 0.46, 0.43, 0.43, 0.43, 0.43, respectively. Additionally, Empirical method gets comparatively a lower score of 0.39, while KDE method has the lowest score of 0.34.

Based on the average precision scores for LUSC dataset that can be seen in Table 4, the ranking of the estimators is as follows: BS, Shrink, Empirical, Adjacency, MM, SG, KDE, Pearson, Kendall and Spearman. BS and Shrink methods could identify the most disease-relevant sub networks with slightly different precision values of 0.76 and 0.75, respectively. Empirical, Adjacency, MM, SG, KDE methods followed them with lower precision scores of 0.68, 0.67, 0.67, 0.64, 0.63, respectively. Additionally, Pearson and Kendall methods obtained comparatively lower scores of 0.59, 0.51, while Spearman method gave the lowest score of 0.46.

According to the average precision scores for SKCM dataset as given in Table 4, the accuracy ranking of the estimators is as follows: MM, Kendall, Shrink, Pearson, Spearman, Adjacency, BS, Empirical, KDE and SG. MM and Kendall methods could identify the most disease-relevant sub networks and genes with slightly different values of 0.758 and 0.757, respectively. Shrink, Pearson, Spearman, Adjacency, BS, Empirical, KDE and SG methods follow them with lower scores of 0.73, 0.71, 0.71, 0.68, 0.68, 0.68, 0.68 and 0.67, respectively.

We designed a 2-level evaluation process, i.e. pathway-level and gene-level, with stringent cut-offs to have a complementary system as explained in the methods section. In pathway-level evaluation we searched the disease-associated modules. Then, in gene-level evaluation we focused on the disease-associated modules and looked for the overlapping ratios between the member genes of these disease-associated modules and the previously reported disease, i.e. given cancer type, genes.

As given in Table 5 and Fig 4, the best performing association estimators according to the average precision values in the gene-level analysis are as follows: Shrink for BRCA, SG for GBM, Shrink for KIRC, BS for LUSC, and MM for SKCM. In addition, as in the pathway-level analysis, although the most successful predictor changes for each data set, the MI-based predictors were found to be more successful in BRCA, GBM, KIRC, LUSC dataset. Only in SKCM data set, MM estimator and Kendall correlation method were found to be successful with very close precision values according to both pathway-level and gene-level analyses for P-value < 0.05.

According to the average precision scores for BRCA dataset, which are given in Table 5, the estimators are listed as follows: Shrink, Empirical, SG, Adjacency, Pearson, MM, Kendall, BS, Spearman, and KDE. Shrink method is the most accurate one in terms of identifying disease-associated genes with a precision score of 0.85. Empirical, SG, Adjacency, Pearson methods follow this one with lower scores of 0.67, 0.63, 0.61, 0.59, respectively. Additionally, MM, Kendall, BS methods obtain comparatively lower scores of 0.52, 0.51, 051, while Spearman and KDE methods get the lowest scores of 0.45, 0.42, respectively.

Based on the average precision scores for GBM dataset that are given in Table 5, the estimators are listed as follows: SG, MM, Kendall, Shrink, BS, KDE, Empirical, Adjacency, Pearson and Spearman. SG is the most accurate method in terms of identifying disease-associated genes with a precision score of 0.80. MM, Kendall, Shrink, BS, KDE methods follow this one with lower scores of 0.72, 0.67, 0.63, 0.62, and 0.60, respectively. Additionally, Empirical, Adjacency and Pearson methods obtain comparatively lower scores of 0.59, 0.58, 0.54, while Spearman method gets the lowest score of 0.46.

According to the average precision scores for KIRC dataset (Table 5), the estimators are listed as follows: Shrink, BS, MM, Pearson, Adjacency, Empirical, Kendall, KDE, Spearman and SG. Shrink is the most accurate method in terms of identifying disease-associated genes with a precision score of 0.51. BS and MM methods follow this one with lower scores of 0.46, 0.43, respectively. Additionally, Pearson, Adjacency, Empirical, Kendall, KDE methods obtain comparatively lower scores of 0.38, 0.37, 0.35, 0.34, 0.34, while Spearman and SG methods have the lowest scores of 0.31 and 0.30.

Based on the average precision scores for LUSC dataset (Table 5), the estimators are listed as follows: BS, Adjacency, Shrink, Empirical, SG, Pearson, MM, KDE, Kendall and Spearman. BS is the most accurate method in terms of identifying disease-associated genes with a precision score of 0.66. Adjacency, Shrink, Empirical, SG methods follow this one with lower scores of 0.63, 0.62, 0.59, 0.59, respectively. Additionally, Pearson, MM, KDE, Kendall methods obtain comparatively lower scores of 0.55, 0.54, 0.46, 0.43, while Spearman method gets the lowest score of 0.38.

According to the average precision scores for SKCM dataset (Table 5), the estimators are listed from high to low scores as follows: MM, Kendall, Shrink, Pearson, Adjacency, BS, Empirical, KDE, SG, and Spearman. MM and Kendall are the most accurate methods in terms of identifying disease-associated genes with precision scores of 0.758 and 0.757, respectively. Shrink, Pearson, Adjacency, BS, Empirical, KDE, and SG methods follow them with lower scores of 0.73, 0.71, 0.68, 0.68, 0.68, 0.68, and 0.67 respectively. Additionally, Spearman method has the lowest score of 0.62.

Finally, the sub networks of the module hub genes identified by the association estimators, which provide the highest precision scores based on a statistical test (with a P-value < 0.05), are generated by Cytoscape [45] and shown in Figs 5–9 for all cancer types for the consideration of the other researchers studying in this field. The genes registered in DisGeNET and experimentally confirmed for the diseases, are shown in Figs 5–9 with colored and larger nodes. Among those, genes that are not colored but have a red frame have a PMID value of one. There is no entry in DisGeNET for the grey colored nodes. Also, the most associated top three biological pathways, to which each module is related, are given above or below the relevant module to annotate each module.

## Conclusion and discussion

Performances of nine association estimators used in the network inference algorithms were examined on the proteomic data of five different cancer types in both pathway and gene-levels. To make this assessment, selected association estimators were used instead of the adjacency matrix construction procedure in the WGCNA, which is based on either Pearson or Spearman correlation and has been used in many different studies in the literature. In conclusion, from Tables 4 and 5 and Figs 3 and 4, it can be clearly observed that in terms of the precision scores, the MI based methods provide better results than the correlation-based methods and the adjacency function which is provided as a default choice in WGCNA. In parallel with the studies in the literature [46], our findings confirmed that the correlation-based methods are not sufficient to estimate the non-linear relationships between the cell molecules such as cancer-related complex molecular interactions.

It was observed that *PDPK1 (PDK1)*, *MSH6*, *BID*, *PECAM1 (CD31)*, *SMAD4*, *GATA3* and *NF2* hub genes (proteins) may have important effects on the breast cancer as a result of the WGCNA analysis with Shrink association estimator. DisGeNET has shown that, among these genes, *MSH6* and *GATA3* were associated with BRCA in only one study, while *RAD50*, *TSC1*, *CHEK2*, *ATM*, *STK11*, *PTEN*, *RAD51*, *BRCA2*, *TPD3*, *CHEK1*, *CCND1*, *ERBB2*, *EGFR*, and *ESR1* were validated in multiple studies. Besides, *PDK1* [47,48] and *SMAD4* [49] genes (proteins) were also shown to be associated with the BRCA in multiple studies, though they were not reported in the DisGeNET.

It was found that *DIRAS3*, *RAD50*, *MAPK1 (ERK2)*, *RBM15*, *IGF1R (IGF1R_pY1135Y1136)*, *MS4A1 (CD20)*, and *COG3* hub genes (proteins) may have important effects on GBM as a result of the WGCNA analysis with SG association estimator. DisGeNET has shown that, among these genes, *IGF1R* was associated with GBM in only one study, while *CHEK2*, *KDR*, *NOTCH1*, *MSH2*, *BCL2*, *ERBB2*, *PDCD4*, *FOXM1*, *PTEN*, *CASP3*, *EGFR*, *TP53BP1*, *IGFBP2*, *RAF1*, *SMAD3*, *SETD2*, *MTOR*, *TP53*, *BRAF*, *MSH6*, *TSC1*, *STAT3*, *MET*, and *PIK3CA* were validated in multiple studies. Besides, in recent studies, *RAD50* [50] gene (protein) was also shown to be associated with the GBM, though it was not reported in the DisGeNET.

It was observed that ADAR (ADAR1), CCNE2 (CYCLINE2), BID, CDKN1B (P27_Pt198), and PRKCA (PKCALPHA) hub genes (proteins) may have significant effects on KIRC as a result of the WGCNA analysis with Shrink association estimator. Thus far, from the DisGeNET, we could not find any evidences that these genes are associated with KIRC. DisGeNET has shown that, ATM, MTOR, TP53, TSC2, ESR1, BCL2, KDR, STAT3, TSC1, and PRKCB were validated in multiple studies.

It was found that *EIF4G1 (EIF4G)*, *FOXM1*, *COPS5 (JAB1)*, *CDKN1A (P21)*, *MRE11A (MRE11)*, and *PRKCB (PKCPANBETAII_pS660)* hub genes (proteins) may have significant effects on LUSC as a result of the WGCNA analysis with BS association estimator. DisGeNET has shown that, among these genes, *EIF4G1 (EIF4G)* gene (protein) was associated with LUSC in two studies, while *CDH1*, *CDKN2A*, *PIK3CA*, *EGFR*, *TP53*, and *STAT3* were confirmed as disease genes in multiple studies. Besides, in recent studies, *FOXM1* [51,52] and *CDKN1A (P21)* [53] genes (proteins) were also shown to be associated with the LUSC in multiple studies though they were not reported in the DisGeNET.

It was observed that *MRE11A (MRE11)*, *ETS1*, *BAX*, *COPS5 (JAB1)*, *TSC2 (TUBERIN_pT1462)*, and *SYK* hub genes (proteins) may have significant effects on SKCM as a result of the WGCNA analysis with MM association estimator. DisGeNET has shown that, *EGFR*, *RAF1*, *PTEN*, *ERCC5*, *CCND1*, *BRCA2*, *PCNA*, *ERBB2*, *TSC1*, *CASP8*, *TP53*, *PIK3CA*, *NRAS*, *BCL2*, *KIT*, *FASN*, *CTNNB1*, *CDKN2A*, *BRAF*, *YAP1*, and *MSH2* were highlighted as disease genes in multiple studies. Besides, in recent studies, *ETS1* [54] gene (protein) was also shown to be associated with the SKCM, though it was not reported in the DisGeNET.

As a conclusion, despite not being included in DisGeNET, the genes that are found to be related with the disease via recent studies in the literature (*PDK1*, *SMAD4*, *RAD50*, *FOXM1*, *CDKN1A*, *ETS1* [47–54]) were detected with our proposed framework. Studies verify that these genes (proteins) are associated with cancer-related processes. Du et al. identified the *PDK1* as a potential therapeutic target for BRCA [47]. Dupuy et al. also determined the *PDK1* as a key regulator of metabolism and metastatic potential in BRCA [48]. Liu at el. indicated that the assessment of *SMAD4* protein level may provide additional prognostic information about BRCA [49]. Mishima et al. found out that *MRE11-RAD50-NBS1* complex inhibitor can effectively increase radiosensitivity in GBM [50]. Sun et al. showed the prognostic significance of *FOXM1* expression in LUSC [51]. Zhang et al. remarked the *FOXM1* as a novel biomarker of LUSC [52]. Fukazawa et al. found out that the tumorigenic effect of *SOX2* on LUSC is mediated in part by suppression of *CDKN1A* [53]. Keehn et al. stated that *ETS1* may be important in the pathogenesis of invasive SKCM [54]. Thus, since our proposed method could capture this long list of previously studied genes, it is suggested that it might capture a more comprehensive list of the disease associated gene-gene interactions that were missed in previous studies.

The most significant contribution of our study is the use of different association estimators in biological network inferring methodologies, which can make a significant improvement in identifying the disease-associated co-expression modules when they are integrated with the WGCNA method. In addition, similar performance scores of each estimator in pathway-level and gene-level analysis also indicate the consistency of our study.

## Supporting information

S1 Fig Number of the modules generated according to the number of genes in the data set for E.coli (Scale-free topology score r^2>0.75). (TIF)

S2 Fig Number of the modules generated according to the number of genes in the data set for Yeast. (Scale-free topology score r^2>0.75).
(TIF)

S3 Fig Average overlapped gene number between DisGeNET and cancer datasets changing by module size. The total overlapped gene numbers in the Data Sets are; BRCA: 15; GBM: 28; LUSC: 7; KIRC: 14; SKCM: 25, respectively.
(TIF)

S1 Table The gene-protein matching. (XLSX)

S2 Table The selected antibodies (proteins) and their expression values' from BRCA dataset. (XLSX)

S3 Table The selected antibodies (proteins) and their expression values' from GBM dataset. (XLSX)

S4 Table The selected antibodies (proteins) and their expression values' from KIRC dataset. (XLSX)

S5 Table The selected antibodies (proteins) and their expression values' from LUSC dataset. (XLSX)

S6 Table The selected antibodies (proteins) and their expression values' from SKCM dataset. (XLSX)

S7 Table The used R packages, functions with parameters and tested values. (DOCX)

S8 Table The parameter values that give the best results according to the dataset in the used functions. (DOCX)

S9 Table P-values (by FET) of association estimators on gene level analysis for BRCA dataset by module size. (XLSX)

S1 Text Module number selection process. (DOCX)

S2 Text Cut-off value selection for the overlap analysis based on Fisher's Exact Test. (DOCX)

## References

[1] Rual J-F, Venkatesan K, Hao T, Hirozane-Kishikawa T, Dricot A, Li N, et al Towards a proteome-scale map of the human protein-protein interaction network. Nature. 2005;437: 1173–1178. Available: doi: 10.1038/nature04209 16189514 10.1038/nature04209

[2] Schadt EE. Molecular networks as sensors and drivers of common human diseases. Nature. Nature Publishing Group; 2009;461: 218–223. Available: doi: https://doi.org/10.1038/nature08454 10.1038/nature08454 19741703

[3] Li J, Lu Y, Akbani R, Ju Z, Roebuck PL, Liu W, et al TCPA: a resource for cancer functional proteomics data. Nat Methods. Nature Publishing Group; 2013;10: 1046–7. doi: 10.1038/nmeth.2650 24037243 10.1038/nmeth.2650 PMC4076789

[4] Siegel R, Naishadham D, Jemal A. Cancer Statistics, 2017. CA Cancer J Clin. 2017;67: 7–30. doi: 10.3322/caac.21387 Available 28055103 10.3322/caac.21387

[5] Olsen C, Meyer PE, Bontempi G. On the impact of entropy estimation on transcriptional regulatory network inference based on mutual information. EURASIP J Bioinform Syst Biol. 2009;2009: 308959 doi: 10.1155/2009/308959 19148299 10.1155/2009/308959 PMC3171423

[6] Margolin AA, Nemenman I, Basso K, Wiggins C, Stolovitzky G, Dalla Favera R, et al ARACNE: an algorithm for the reconstruction of gene regulatory networks in a mammalian cellular context. BMC Bioinformatics. 2006;7 Suppl 1: S7 doi: 10.1186/1471-2105-7-S1-S7 16723010 10.1186/1471-2105-7-S1-S7 PMC1810318

[7] Faith JJ, Hayete B, Thaden JT, Mogno I, Wierzbowski J, Cottarel G, et al Large-scale mapping and validation of Escherichia coli transcriptional regulation from a compendium of expression profiles. PLoS Biol. 2007;5: e8 doi: 10.1371/journal.pbio.0050008 17214507 10.1371/journal.pbio.0050008 PMC1764438

[8] Peng H, Long F, Ding C. Feature selection based on mutual information: criteria of max-dependency, max-relevance, and min-redundancy. IEEE Trans Pattern Anal Mach Intell. 2005;27: 1226–38. doi: 10.1109/TPAMI.2005.159 16119262 10.1109/TPAMI.2005.159

[9] de Matos Simoes R, Emmert-Streib F. Influence of statistical estimators of mutual information and data heterogeneity on the inference of gene regulatory networks. PLoS One. 2011;6 doi: 10.1371/journal.pone.0029279 22242113 10.1371/journal.pone.0029279 PMC3248437

[10] Altay G, Emmert-Streib F. Inferring the conservative causal core of gene regulatory networks. BMC Syst Biol. 2010;4: 132 doi: 10.1186/1752-0509-4-132 20920161 10.1186/1752-0509-4-132 PMC2955605

[11] Daub CO, Steuer R, Selbig J, Kloska S, Schena M, Shalon D, et al Estimating mutual information using B-spline functions–an improved similarity measure for analysing gene expression data. BMC Bioinformatics. 2004;5: 118 doi: 10.1186/1471-2105-5-118 15339346 10.1186/1471-2105-5-118 PMC516800

[12] Kurt Z, Aydin N, Altay G. A comprehensive comparison of association estimators for gene network inference algorithms. Bioinformatics. 2014;30: 2142–2149. doi: 10.1093/bioinformatics/btu182 24728859 10.1093/bioinformatics/btu182

[13] Şenbabaoğlu Y, Sümer O, Ciriello G, Schultz N, Sander C. A Multi-Method Approach for Proteomic Network Inference in 11 Human Cancers. PLoS Comput Biol. 2016;12: 1–31. doi: 10.1371/journal.pcbi.1004765 26928298 10.1371/journal.pcbi.1004765 PMC4771175

[14] Madhamshettiwar PB, Maetschke SR, Davis MJ, Reverter A, Ragan MA. Gene regulatory network inference: evaluation and application to ovarian cancer allows the prioritization of drug targets. Genome Med. 2012;4: 41 doi: 10.1186/gm340 22548828 10.1186/gm340 PMC3506907

[15] Langfelder P, Horvath S. WGCNA: an R package for weighted correlation network analysis. BMC Bioinformatics. 2008;9: 559 doi: 10.1186/1471-2105-9-559 19114008 10.1186/1471-2105-9-559 PMC2631488

[16] Horvath S, Zhang B, Carlson M, Lu K V, Zhu S, Felciano RM, et al Analysis of oncogenic signaling networks in glioblastoma identifies ASPM as a molecular target. Proc Natl Acad Sci. 2006;103: 17402–17407. doi: 10.1073/pnas.0608396103 17090670 10.1073/pnas.0608396103 PMC1635024

[17] Carlson MRJ, Zhang B, Fang Z, Mischel PS, Horvath S, Nelson SF. Gene connectivity, function, and sequence conservation: predictions from modular yeast co-expression networks. BMC Genomics. London: BioMed Central; 2006;7: 40 doi: 10.1186/1471-2164-7-40 16515682 10.1186/1471-2164-7-40 PMC1413526

[18] Fuller TF, Ghazalpour A, Aten JE, Drake TA, Lusis AJ, Horvath S. Weighted gene coexpression network analysis strategies applied to mouse weight. Mamm Genome. 2007;18: 463–472. doi: 10.1007/s00335-007-9043-3 17668265 10.1007/s00335-007-9043-3 PMC1998880

[19] Emilsson V, Thorleifsson G, Zhang B, Leonardson AS, Zink F, Zhu J, et al Genetics of gene expression and its effect on disease. Nature. Nature Publishing Group; 2008;452: 423–428. doi: 10.1007/s00335-007-9043-3 10.1038/nature06758 18344981

[20] Keller MP, Choi Y, Wang P, Belt Davis D, Rabaglia ME, Oler AT, et al A gene expression network model of type 2 diabetes links cell cycle regulation in islets with diabetes susceptibility. Genome Res. Cold Spring Harbor Laboratory Press; 2008;18: 706–716. doi: 10.1101/gr.074914.107 18347327 10.1101/gr.074914.107 PMC2336811

[21] Ruyssinck J, Huynh-Thu VA, Geurts P, Dhaene T, Demeester P, Saeys Y. NIMEFI: Gene Regulatory Network Inference using Multiple Ensemble Feature Importance Algorithms. PLoS One. 2014;9: e92709 doi: 10.1371/journal.pone.0092709 24667482 10.1371/journal.pone.0092709 PMC3965471

[22] Akbani R, Becker K-F, Carragher N, Goldstein T, de Koning L, Korf U, et al Realizing the promise of reverse phase protein arrays for clinical, translational, and basic research: a workshop report: the RPPA (Reverse Phase Protein Array) society. Mol Cell Proteomics. 2014;13: 1625–43. doi: 10.1074/mcp.O113.034918 24777629 10.1074/mcp.O113.034918 PMC4083105

[23] Sheehan KM, Calvert VS, Kay EW, Lu Y, Fishman D, Espina V, et al Use of Reverse Phase Protein Microarrays and Reference Standard Development for Molecular Network Analysis of Metastatic Ovarian Carcinoma. Mol Cell Proteomics. 2005;4: 346–355. doi: 10.1074/mcp.T500003-MCP200 15671044 10.1074/mcp.T500003-MCP200

[24] The University of Texas MD Anderson Cancer Center. TCPA: Home [Internet]. 2013 [cited 21 May 2017]. Available: http://www.tcpaportal.org/tcpa/

[25] Piñero J, Bravo À, Queralt-Rosinach N, Gutiérrez-Sacristán A, Deu-Pons J, Centeno E, et al DisGeNET: a comprehensive platform integrating information on human disease-associated genes and variants. Nucleic Acids Res. 2016;45: gkw943 doi: 10.1093/nar/gkw943 27924018 10.1093/nar/gkw943 PMC5210640

[26] Piñero J, Queralt-Rosinach N, Bravo À, Deu-Pons J, Bauer-Mehren A, Baron M, et al DisGeNET: a discovery platform for the dynamical exploration of human diseases and their genes. Database J Biol Databases Curation. Oxford University Press; 2015;2015: bav028 doi: 10.1093/database/bav028 25877637 10.1093/database/bav028 PMC4397996

[27] Subramanian A, Tamayo P, Mootha VK, Mukherjee S, Ebert BL, Gillette M a, et al Gene set enrichment analysis: a knowledge-based approach for interpreting genome-wide expression profiles. Proc Natl Acad Sci U S A. 2005;102: 15545–50. doi: 10.1073/pnas.0506580102 16199517 10.1073/pnas.0506580102 PMC1239896

[28] McDonald JH. Handbook of Biological Statistics. Sparky House Publ. 2009; 291 doi: 10.1017/CBO9781107415324.004

[29] Kendall MG (Maurice G, Gibbons Jean Dickinson 1938- Rank correlation methods. 5th ed. London: Edward Arnold; 1990.

[30] Paninski L. Estimation of Entropy and Mutual Information. Neural Comput. Cambridge, MA, USA: MIT Press; 2003;15: 1191–1253. doi: 10.1162/089976603321780272

[31] Moon Y-I, Rajagopalan B, Lall U. Estimation of mutual information using kernel density estimators. Phys Rev E. American Physical Society; 1995;52: 2318–2321. Available: https://link.aps.org/doi/10.1103/PhysRevE.52.2318 10.1103/physreve.52.2318 9963673

[32] Miller GA. Note on the bias of information estimates. Information Theory in Psychology: Problems and Methods. 1955 pp. 95–100.

[33] Hausser J, Strimmer K. Entropy inference and the James-Stein estimator, with application to nonlinear gene association networks. J Mach Learn Res. 2009;10: 1469–1484. Available: http://arxiv.org/abs/0811.3579

[34] Schurmann, Thomas Grassberger P. Entropy estimation of symbol sequences. Chaos An Interdiscip J Nonlinear Sci. American Institute of Physics; 1996;6: 414–427. doi: 10.1063/1.166191 12780271 10.1063/1.166191

[35] Meyer PE, Lafitte F, Bontempi G. minet: A R/Bioconductor Package for Inferring Large Transcriptional Networks Using Mutual Information. BMC Bioinformatics. 2008;9: 461 doi: 10.1186/1471-2105-9-461 18959772 10.1186/1471-2105-9-461 PMC2630331

[36] Ravasz E, Somera AL, Mongru DA, Oltvai ZN, Barabási A-L. Hierarchical Organization of Modularity in Metabolic Networks. Science (80-). 2002;297: 1551 LP-1555. Available: http://science.sciencemag.org/content/297/5586/1551.abstract 10.1126/science.1073374 12202830

[37] Yip AM, Horvath S. Gene network interconnectedness and the generalized topological overlap measure. BMC Bioinformatics. 2007;8: 22 doi: 10.1186/1471-2105-8-22 17250769 10.1186/1471-2105-8-22 PMC1797055

[38] Langfelder P, Zhang B, Horvath S. Defining clusters from a hierarchical cluster tree: the Dynamic Tree Cut package for R. Bioinformatics. 2008;24: 719 doi: 10.1093/bioinformatics/btm563 18024473 10.1093/bioinformatics/btm563

[39] Fisher RAS. Statistical methods for research workers [Internet]. 7th ed., r. Edinburgh Oliver and Boyd; 1938 Available: http://openlibrary.org/books/OL181269M

[40] Fury W, Batliwalla F, Gregersen PK, Li W. Overlapping Probabilities of Top Ranking Gene Lists, Hypergeometric Distribution, and Stringency of Gene Selection Criterion. Engineering in Medicine and Biology Society, 2006 EMBS ‘06 28th Annual International Conference of the IEEE. 2006 pp. 5531–5534. doi: 10.1109/IEMBS.2006.260828 10.1109/IEMBS.2006.260828 17947148

[41] Ezkurdia I, Juan D, Rodriguez JM, Frankish A, Diekhans M, Harrow J, et al Multiple evidence strands suggest that there may be as few as 19 000 human protein-coding genes. Hum Mol Genet. 2014;23: 5866–5878. Available: doi: 10.1093/hmg/ddu309 24939910 10.1093/hmg/ddu309 PMC4204768

[42] Altay G, Kurt Z, Altay N, Aydin N. DepEst: an R package of important dependency estimators for gene network inference algorithms. bioRxiv. 2017;1.

[43] Integrative Biomedical Informatics Group GRIB/IMIM/UPF. DisGeNET—WEB [Internet]. [cited 14 May 2017]. Available: http://www.disgenet.org/web/DisGeNET/menu

[44] GSEA and MSigDB Team. GSEA | MSigDB [Internet]. 2015 [cited 21 May 2017]. Available: http://software.broadinstitute.org/gsea/msigdb/index.jsp

[45] Christmas Rowan; Avila-Campillo Iliana; Bolouri Hamid; Schwikowski Benno; Anderson Mark; Kelley Ryan; Landys Nerius; Workman Chris; Ideker Trey; Cerami Ethan; Sheridan Rob; Bader Gary D.; Sander C. Cytoscape: a software environment for integrated models of biomolecular interaction networks. Am Assoc Cancer Res Educ B. 2005; 12–16. doi: 10.1101/gr.1239303.metabolite

[46] Song L, Langfelder P, Horvath S. Comparison of co-expression measures: mutual information, correlation, and model based indices. BMC Bioinformatics. 2012;13: 328 doi: 10.1186/1471-2105-13-328 23217028 10.1186/1471-2105-13-328 PMC3586947

[47] Du J, Yang M, Chen S, Li D, Chang Z, Dong Z. PDK1 promotes tumor growth and metastasis in a spontaneous breast cancer model [Internet]. Oncogene. Macmillan Publishers Limited; 2016 pp. 3314–3323. Available: doi: 10.1038/onc.2015.393 26455327 10.1038/onc.2015.393

[48] Dupuy F, Tabariès S, Andrzejewski S, Dong Z, Blagih J, Annis MG, et al PDK1-Dependent Metabolic Reprogramming Dictates Metastatic Potential in Breast Cancer. Cell Metab. Elsevier; 2017;22: 577–589. doi: 10.1016/j.cmet.2015.08.007 10.1016/j.cmet.2015.08.007 26365179

[49] LIU N, YU C, SHI Y, JIANG J, LIU Y. SMAD4 expression in breast ductal carcinoma correlates with prognosis. Oncol Lett. D.A. Spandidos; 2015;10: 1709–1715. doi: 10.3892/ol.2015.3442 26622737 10.3892/ol.2015.3442 PMC4533521

[50] Mishima K, Mishima-Kaneko M, Kawata T, Saya H, Ishimaru N, Yamada K, et al MRE11-RAD50-NBS1 COMPLEX INHIBITOR MIRIN ENHANCES RADIOSENSITIVITY IN HUMAN GLIOBLASTOMA CELLS. Neuro Oncol. Oxford University Press; 2014;16: iii36–iii36. doi: 10.1093/neuonc/nou208.51

[51] Sun Q, Dong M, Chen Y, Zhang J, Qiao J, Guo X. Prognostic significance of FoxM1 expression in non-small cell lung cancer. J Thorac Dis. AME Publishing Company; 2016;8: 1269–1273. doi: 10.21037/jtd.2016.04.13 27293846 10.21037/jtd.2016.04.13 PMC4885990

[52] Zhang J, Zhang J, Cui X, Yang Y, Li M, Qu J, et al FoxM1: a novel tumor biomarker of lung cancer. Int J Clin Exp Med. e-Century Publishing Corporation; 2015;8: 3136–3140. Available: http://www.ncbi.nlm.nih.gov/pmc/articles/PMC4443037/ 26064203 PMC4443037

[53] Fukazawa T, Guo M, Ishida N, Yamatsuji T, Takaoka M, Yokota E, et al SOX2 suppresses CDKN1A to sustain growth of lung squamous cell carcinoma. Sci Rep. Nature Publishing Group; 2016;6: 20113 doi: 10.1038/srep20113 26846300 10.1038/srep20113 PMC4742851

[54] Keehn CA, Smoller BR, Morgan MB. Ets-1 immunohistochemical expression in non-melanoma skin carcinoma. J Cutan Pathol. Munksgaard International Publishers; 2004;31: 8–13. doi: 10.1046/j.0303-6987.2004.0158.x 14675279 10.1046/j.0303-6987.2004.0158.x

