# Supplementary figures and images for: Estimation of the proteomic cancer co-expression sub networks by using association estimators
Source: PLoS One. 2017 Nov 16;12(11):e0188016. doi: 10.1371/journal.pone.0188016 (PMC5690670; doi:10.1371/journal.pone.0188016)

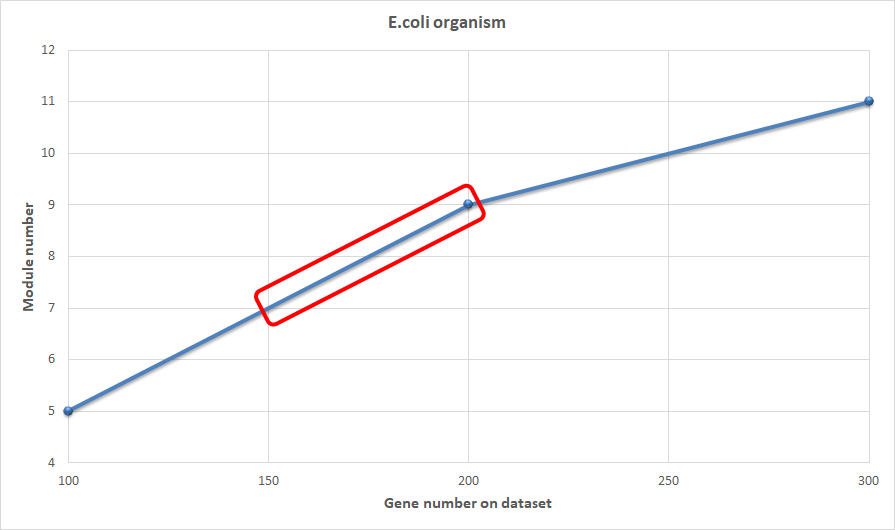

Supplement: S1 Fig — (TIF) [file pone.0188016.s001.tif]

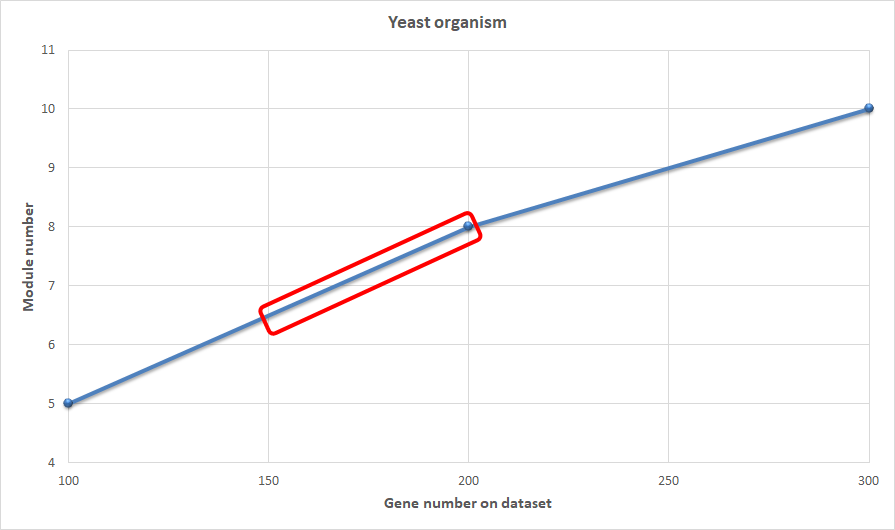

Supplement: S2 Fig — (Scale-free topology score r^2>0.75). (TIF) [file pone.0188016.s002.tif]

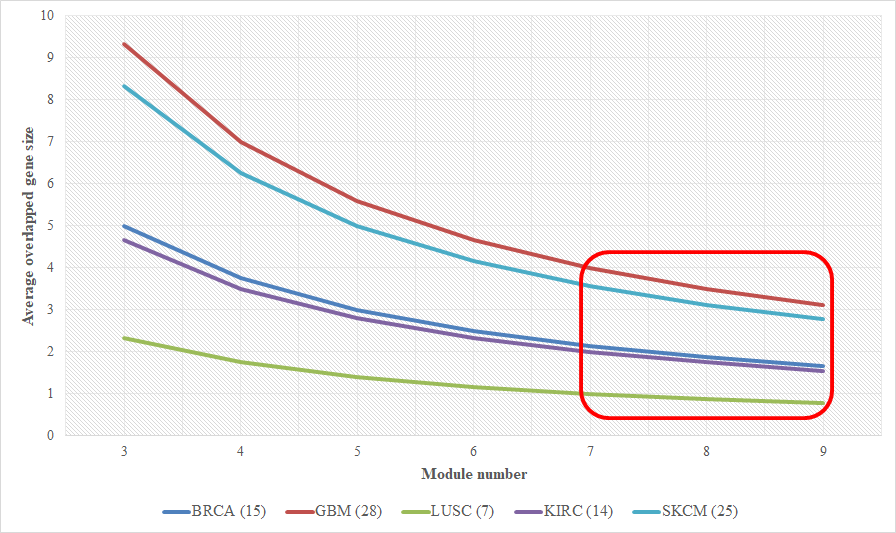

Supplement: S3 Fig — The total overlapped gene numbers in the Data Sets are; BRCA: 15; GBM: 28; LUSC: 7; KIRC: 14; SKCM: 25, respectively. (TIF) [file pone.0188016.s003.tif]
