# Supplementary material for: Estimation of the proteomic cancer co-expression sub networks by using association estimators
Source: PLoS One. 2017 Nov 16;12(11):e0188016. doi: 10.1371/journal.pone.0188016 (PMC5690670; doi:10.1371/journal.pone.0188016)
Supplement: S7 Table — (DOCX) [file pone.0188016.s010.docx]

S7 Table

S7 Table shows the R packages we used for the analysis operations and the functions used in these packages together with their parameters. The description of the used functions can be found in WGCNA [1], minet [2] and DepEst [3] R packages.

**S7 Table** The used R packages, functions with parameters and tested values

| **Packages** | **Functions** | **Parameters** | **Tested values** |
| --- | --- | --- | --- |
| minet | *build.mim* | estimator | “spearman”, “pearson”, “kendall”, “mi. empirical”, “mi.mm”, “mi.shrink”, and “mi.sg”. |
|  |  | disc | “none”, “equalfreq”, “equalwidth” and “globalequalwidth”. |
| DepEst | *obtain.mim* | estimator | “b.spline”, “KDE”. |
|  |  | cop.transform | TRUE, FALSE. |
| WGCNA | *TOMsimilarity* | TOMDenom | min, mean. |
|  | *hclust* | method | “ward.D”, “ward.D2”, “single”, “complete”, “average”, “mcquitty”. |
|  | *cutreeDynamic* | deepSplit | 0 to 4 increasing by 1. |
|  |  | minClusterSize | 10 to 20 increasing by 1. |
|  |  | cutHeight | 0.7 to 0.999 increasing by 0.001. |

# References

1. Langfelder P, Horvath S. WGCNA: an R package for weighted correlation network analysis. BMC Bioinformatics. 2008;9: 559. doi:10.1186/1471-2105-9-559

2. Meyer PE, Lafitte F, Bontempi G. minet: A R/Bioconductor Package for Inferring Large Transcriptional Networks Using Mutual Information. BMC Bioinformatics. 2008;9: 461. doi:10.1186/1471-2105-9-461

3. Altay G, Kurt Z, Altay N, Aydin N. DepEst: an R package of important dependency estimators for gene network inference algorithms. bioRxiv. 2017;1.
