# Supplementary material for: Estimation of the proteomic cancer co-expression sub networks by using association estimators
Source: PLoS One. 2017 Nov 16;12(11):e0188016. doi: 10.1371/journal.pone.0188016 (PMC5690670; doi:10.1371/journal.pone.0188016)
Supplement: S8 Table — (DOCX) [file pone.0188016.s011.docx]

S8 Table

As a result of our work, according to dataset the most successful association estimators and the parameter values of the used functions are given in S9 Table.

**S8 Table** The Parameter values that give the best results according to the dataset in the used functions.

|  |  | **Functions, Parameters and Values** | | | | | | | | |
| --- | --- | --- | --- | --- | --- | --- | --- | --- | --- | --- |
| Module  Size | Dataset | build.mim | | obtain.mim | | TOMsimilarity | hclust | cutreeDynamic | | |
|  |  | Estimator | discretization method | Estimator | cop.transform | TOMDenom | method | min Cluster Size | cut Height | deep Split |
| 7 | BRCA | Shrink | globalequalwidth | - | - | mean | ward.D2 | 14 | 0.994 | 2 |
| 8 | BRCA | Shrink | equalfreq | - | - | min | complete | 10 | 0.995 | 2 |
| 9 | BRCA | Shrink | globalequalwidth | - | - | mean | ward.D2 | 10 | 0.986 | 2 |
| 7 | GBM | SG | equalfreq | - | - | mean | ward.D | 13 | 0.887 | 1 |
| 8 | GBM | SG | globalequalwidth | - | - | mean | ward.D2 | 12 | 0.991 | 3 |
| 9 | GBM | SG | globalequalwidth | - | - | mean | ward.D2 | 11 | 0.991 | 3 |
| 7 | KIRC | Shrink | equalfreq | - | - | min | complete | 14 | 0.995 | 3 |
| 8 | KIRC | Shrink | equalfreq | - | - | min | complete | 11 | 0.995 | 3 |
| 9 | KIRC | Shrink | equalfreq | - | - | min | complete | 10 | 0.994 | 4 |
| 7 | LUSC | - | - | BS | TRUE | mean | ward.D2 | 10 | 0.848 | 2 |
| 8 | LUSC | - | - | BS | TRUE | mean | ward.D | 10 | 0.866 | 2 |
| 9 | LUSC | - | - | BS | TRUE | mean | ward.D | 11 | 0.866 | 3 |
| 7 | SKCM | MM | equalfreq | - | - | min | complete | 13 | 0.945 | 1 |
| 8 | SKCM | MM | equalwidth | - | - | min | complete | 10 | 0.854 | 0 |
| 9 | SKCM | MM | globalequalwidth | - | - | min | complete | 10 | 0.985 | 1 |

As can be seen in S8 Table, the association estimator is changed according to the data set. Also the discretization method, the hierarchical clustering method and the method of calculating the topological overlap matrix are also changed. The distributions of these methods according to successful results are given in Figs 2-4.


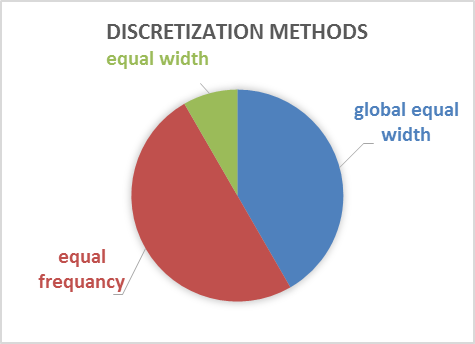


**Fig 1.** Distributions of discretization methods used in association estimators that are successful in the analysis.


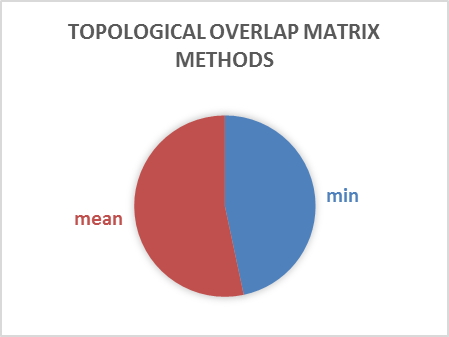


**Fig 2.** Distributions of Topological overlap matrix method used in association estimators that are successful in the analysis.


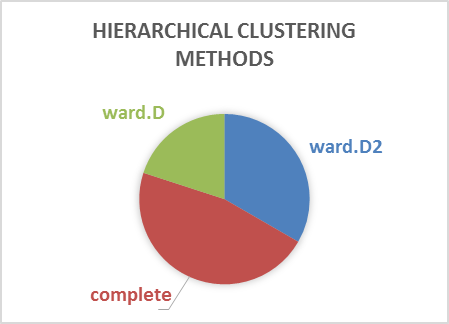


**Fig 3.** Distributions of hierarchical clustering method used in association estimators that are successful in the analysis.
