# Supplementary material for: Estimation of the proteomic cancer co-expression sub networks by using association estimators
Source: PLoS One. 2017 Nov 16;12(11):e0188016. doi: 10.1371/journal.pone.0188016 (PMC5690670; doi:10.1371/journal.pone.0188016)
Supplement: S1 Text — (DOCX) [file pone.0188016.s013.docx]

**S1 Text**

Module number selection process

In the literature, the data sets analyzed by WGCNA contain approximately 1000 or more genes/proteins and these genes/proteins are clustered in different number of modules. For example, a data set with 862 proteins from Drosophila were clustered in 28 modules, and 2050 proteins from the Yeast dataset were clustered in 44 modules [1]. The number of genes/proteins within the data sets analyzed in our study varies between 169 and 173, and these gene numbers are very small compared to that of many studies in the literature. So we followed the steps that are listed below to determine the module numbers according to the size of the dataset.

1. Firstly, we produced simulated gene expression datasets using SynTReN [2], which is a network generator that produces artificial gene expression data that approximates experimental data, from e.coli and yeast organisms. Properties of the generated data sets and the SynTReN parameters used in the data set production are given in Tables 1 and 2, respectively.

**Table 1.** **Properties of the generated data sets.**

| Dataset | Topology | Experiments | Gene Number |
| --- | --- | --- | --- |
| ecoli100 | e.coli | 200 | 100 |
| ecoli200 |  |  | 200 |
| ecoli300 |  |  | 300 |
| yeast100 | yeast |  | 100 |
| yeast200 |  |  | 200 |
| yeast300 |  |  | 300 |

We used SynTReN version 1.2 on gene expression data production.

**Table 2. SynTReN parameters used in the dataset generation.**

| **Parameter name** | **Value** |
| --- | --- |
| BurnIn period | 1000 |
| Number of experiments | 200 |
| Number of samples per experiment | 1 |
| Number of nodes | 100 to 300 increasing by 100 |
| Number of background nodes | 0 |
| Probability for complex 2-regulator interactions | 0.3 |
| Biological noise | 0.1 |
| Experimental noise | 0.1 |
| Noise on correlated inputs | 0.1 |
| Number of external nodes | -1 |
| Number of correlated external nodes | -1 |
| Subnetwork selection method | neighbor addition |
| Random seed | 13 |

1. Secondly, we analyzed the gene expression datasets generated on the previous step by using the default parameter settings of the WGCNA R package. According to this analysis, we found out that the datasets with about 150-200 genes produced 7-8-9 modules with a scale-free topology. We used the scale-free topology-related r^2 score, which is reported by the WGCNA tool, to evaluate whether the network exhibits a scale free topology. A score r^2>0.75 would mean that the network topology is scale-free as in many biological networks [3]. Also as the number of genes in the data set increases, the number of modules increases to reach the same scale-free topology cut-off. In order to better illustrate our approach to determine the number of modules, we have added Supplemental Figures (S1 Fig - S2 Fig) below.


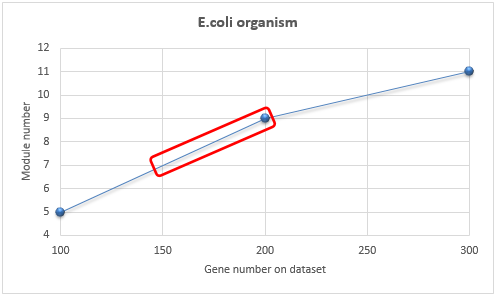


**S1 Fig. Number of the modules generated according to the number of genes in the data set for E.coli (Scale-free topology score r^2>0.75).**


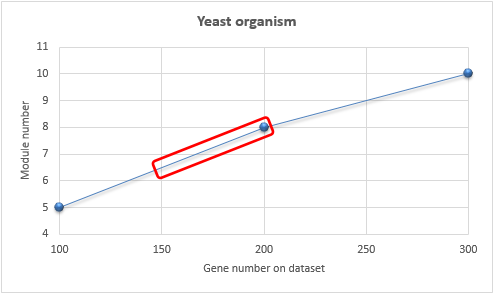


**S2 Fig. Number of the modules generated according to the number of genes in the data set for Yeast. (Scale-free topology score r^2>0.75).**

As shown in S1 Fig, we selected module numbers 7-8-9 to evaluate the association estimators’ performances on cancer datasets, since the data sets which were analyzed in our study contained 169-173 proteins.

**References**

1. Dong J, Horvath S. Understanding network concepts in modules. BMC Syst Biol. BioMed Central; 2007;1: 24. doi:10.1186/1752-0509-1-24

2. Van den Bulcke T, Van Leemput K, Naudts B, van Remortel P, Ma H, Verschoren A, et al. SynTReN: a generator of synthetic gene expression data for design and analysis of structure learning algorithms. BMC Bioinformatics. London: BioMed Central; 2006;7: 43. doi:10.1186/1471-2105-7-43

3. Langfelder P, Horvath S. WGCNA: an R package for weighted correlation network analysis. BMC Bioinformatics. 2008;9: 559. doi:10.1186/1471-2105-9-559
