# Supplementary material for: Estimation of the proteomic cancer co-expression sub networks by using association estimators
Source: PLoS One. 2017 Nov 16;12(11):e0188016. doi: 10.1371/journal.pone.0188016 (PMC5690670; doi:10.1371/journal.pone.0188016)
Supplement: S2 Text — (DOCX) [file pone.0188016.s014.docx]

**S2 Text**

Cut-off value selection for the overlap analysis based on Fisher's Exact Test

Our rationale to assume that a module is a hit (a disease-associated module) if there are at least two disease-associated pathways, which both share at least five genes with this module, is to increase the accuracy of our statistical and computational analysis. As seen in S3 Fig the average overlapped gene number between DisGeNET genes and the cancer protein datasets are changing between 1 and 4 when we have a module number changing between 7 and 9. So, choosing 5 as a cut-off for evaluating the overlapping ratio is a more stringent cut-off then average shared gene numbers (i.e. 1-4). Based on this analysis, the detection of disease-related genes with the proposed framework indicates the stringency and accuracy of our selection criteria based on these cut-off values.

**S3 Fig. Average Overlapped Gene Number between DisGeNET and Cancer Datasets changing by Module Size.** The total overlapped gene numbers in the Data Sets are; BRCA: 15; GBM: 28; LUSC: 7; KIRC: 14; SKCM: 25, respectively.
